# Digital Tools’ Effectiveness on Physical Activity Outcomes in Children and Adolescents: Umbrella Review

**DOI:** 10.2196/75769

**Published:** 2026-03-24

**Authors:** Garden Tabacchi, Antonino Scardina, Antonella Amato, Marta Giardina, Giulia Accardi, Valentina Di Liberto, Giuseppe Giglia, Sonya Vasto, Monica Frinchi, Paolo Boffetta, Walter Mazzucco, Marianna Bellafiore

**Affiliations:** 1 Department of Psychology, Educational Science and Human Movement, University of Palermo, Via Giovanni Pascoli 6, Palermo, PA, 90144, Italy, 39 09123896917; 2 Department of Biological, Chemical and Pharmaceutical Sciences and Technologies, University of Palermo, Palermo, PA, Italy; 3 Department of Biomedicine, Neuroscience and Advanced Diagnostics (BIND), University of Palermo, Palermo, PA, Italy; 4 Department of Medicine and Surgery, Università degli Studi di Enna Kore, Enna, Sicily, Italy; 5 Department of Medical and Surgical Sciences, University of Bologna, Bologna, Italy

**Keywords:** physical activity, digital, effectiveness, children, adolescents, mobile phone

## Abstract

**Background:**

A substantial proportion of children and adolescents worldwide do not meet current physical activity (PA) guidelines. So digital tools interventions have been implemented worldwide. However, evidence regarding the effectiveness of these remains inconsistent, underscoring the need for a comprehensive synthesis of the available literature.

**Objective:**

This umbrella review aimed to summarize and critically evaluate the characteristics and effectiveness of digital interventions designed to increase PA in children and adolescents.

**Methods:**

An overview of systematic reviews (SRs) and meta-analyses of randomized controlled trials (RCTs) was conducted in accordance with the PRIOR (Preferred Reporting Items for Overviews of Reviews) and the PRISMA-S (Preferred Reporting Items for Systematic Reviews and Meta-Analyses Literature Search Extension) checklists. Reviews published between 2018 and 2025 were searched in SCOPUS, PubMed or MEDLINE, Web of Science, Cochrane Database of Systematic Reviews, and SPORTDiscus or EBSCO, using a combination of terms addressing the type of digital tool (eg, apps, wearables, etc) and device-based PA outcomes. Eligible SRs and meta-analyses focused on populations aged 6‐17 years and evaluated digital interventions aimed at increasing PA. Methodological quality was assessed using AMSTAR 2 (A Measurement Tool to Assess Systematic Reviews) for SRs and meta-analyses, and Risk of Bias 2 for RCTs. Intervention characteristics and effectiveness outcomes were summarized as frequencies, and chi-square tests were applied to explore differences in effectiveness across study features.

**Results:**

Forty-eight SRs or meta-analyses comprising 62 RCTs were included. The reviews’ quality was moderate to poor, and 7/62 (11.3%) of RCTs were judged to have a high risk of bias. The mainly addressed PA outcomes were moderate-to-vigorous PA and step counts, commonly measured using accelerometers and pedometers. Overall, 45.2% of interventions demonstrated effectiveness for at least 1 PA outcome. Higher effectiveness rates were observed in RCTs that targeted PA as the sole primary outcome (19/32, 59.4%), used wearables as both the digital intervention component (7/11, 63.6%) and delivery device (8/12, 66.7%), and used pedometers for PA outcome measurement (13/16, 81.3%). Significant differences in effectiveness were found for the type of PA assessment device (*P*=.003) and for interventions targeting low-income populations, which showed lower effectiveness (*P*=.01). Additional trends were identified for geographic region (*P*=.06), intervention setting (*P*=.09), baseline activity level (*P*=.06), intervention focus (*P*=.09), and device brand (*P*=.09).

**Conclusions:**

This novel umbrella review provides a comprehensive synthesis of digital PA interventions in youth, foreseeing potential factors that may influence their effectiveness, and highlighting methodological limitations. It offers evidence-based insights for practitioners, educators, and policymakers, helping to identify digital tools most likely to successfully increase PA in youth. Future research should prioritize stronger methodological rigor and more precise intervention designs. This has clear value for the public health practice to reduce long-term disease risk.

## Introduction

Physical activity (PA) is an important determinant of health status. Nearly a third of the population in the world does not meet the PA recommended levels [1], and this can be observed already in young ages, with 80% of adolescents aged 11‐17 years not meeting the current PA guidelines [2]. This low PA level in children and adolescents may lead to obesity, poor cardiometabolic health, low fitness levels, sleep disturbances, and other diseases in adulthood.

Continuous monitoring of PA is thus needed to identify trends and priorities in public health, and effective interventions are mandatory to improve PA in youngsters and prevent future health status impairment. Among the most common actions, increasing evidence outlines the use of digital interventions in youth to improve PA and reduce sedentary behaviors (SBs), beyond addressing other health-related attitudes such as eating, tobacco smoking, or alcohol drinking.

Recently, the Global Action Plan on Physical Activity 2018‐2030 of the World Health Organization underlined the need to increase PA by 15% by 2030, through different policy actions that also include digital approaches such as apps, wearables, or virtual coaching [3,4].

Digital technologies are particularly appealing to children and adolescents, and this is supported by recent data indicating that among those aged 15 years, more than 50% spent 30+ hours/week using digital devices, and in some countries up to 43% spent 60+ hours/week [5]. The main used technology is the internet, with 1 in 3 internet users in the world being children and adolescents [6], and 98% of those aged 15 years having a smartphone with internet connection [5]. The main digital devices used are smartphones, with the rate of adoption having increased particularly in those of young ages; 95% of teens from the US and 86% of European adolescents use smartphones and social media every day [5,7]. Other digital devices used in this age can be PCs, tablets, smartwatches, activity trackers, virtual reality headsets, and other wearables, and digital components can include websites, apps, text messages, voice messages, gamification components, virtual coaching, virtual reality, etc.

As all these tools can offer effective ways to improve and promote PA in youth, they have been increasingly included in tailored interventions in recent years [8,9]. These interventions were conducted in different settings, including education contexts such as schools, although only 40% of primary schools, 50% of lower secondary schools, and 65% of upper secondary schools were connected to the internet for pedagogical purposes [10]. Several studies were found on behavior change techniques (ie, activity monitoring with feedback, goal setting, graded tasks, or social incentives) [11-13], which are supposed to be effective for initiating and maintaining active behaviors over time by enhancing motivation and accountability.

Nonetheless, still clearer information is needed on the effectiveness of digital approaches in modifying PA behaviors of young populations. Literature shows that interventions aimed at increasing PA have been performed in different ways, with different designs and digital tools [14]. Thus, the main objective of this umbrella review is to systematically summarize and critically evaluate the characteristics and effectiveness of digital interventions aimed at increasing PA in children and adolescents, with particular attention to intervention components, determinants of effectiveness, and methodological limitations.

## Methods

### Protocol and Registration

This umbrella review was registered in the PROSPERO (International Prospective Register of Systematic Reviews; CRD42024510602). The search strategy was amended from what was originally specified at registration and in the protocol. The search was performed by following the PRIOR (Preferred Reporting Items for Overviews of Reviews) [15] and the PRISMA-S (Preferred Reporting Items for Systematic Review and Meta-Analyses Literature Search Extension) [16] checklists (Checklists1  2).

### Study Design

The present review is part of a larger systematic review (SR) protocol, which includes studies identifying digital interventions aimed to improve different lifestyles, such as PA, dietary behaviors, migraine, or sleep patterns, within the DARE (Digital Lifelong Prevention) Project. The DARE Project is an initiative funded by the Italian Ministry of Universities and Research within the National Recovery and Resilience Plan, aimed at using digital technologies to create a data-driven health care ecosystem that can contribute to promoting healthy lifestyles and disease prevention and cure.

A systematically conducted overview of reviews and meta-analyses was chosen as the best design for the search and is hereafter referred to as an umbrella review. An umbrella review can be viewed as a way to obtain evidence from different existing reviews that address a broader question and can be useful for comparing interventions and developing guidelines, thus providing a very high level of evidence [17,18]. As a huge number of studies have been conducted and published in the literature on this topic, most of them lacking methodological rigor, the present search was limited to capturing SRs and meta-analyses that collected experimental studies, that is, randomized controlled trials (RCTs). SRs included by the authors followed a strict, peer-reviewed protocol to ensure all available evidence was considered impartially, involving a comprehensive literature search, critical appraisal of study quality, and statistical synthesis of results to provide a clear, evidence-based answer.

### Search Strategy

The review was conducted on 5 databases: Scopus, PubMed or MEDLINE, Web of Science, the Cochrane Database of Systematic Reviews via the Cochrane Library, and SPORTDiscus via EBSCOhost. The search was conducted in November 2025, with record publication dates restricted to the years 2018‐2025, informed by evidence that research on second-generation technologies (such as smartphones and wearables) has risen sharply since 2013 [19]; a 2018 review would therefore be expected to encompass a larger set of previously used tools.

A manual search was conducted in parallel by browsing the reference lists of all included reviews, by searching for information on the websites, and from gray literature. For papers not freely available on the internet, the authors were contacted and asked to provide the full-text versions.

Keywords used for the search included a combination of terms addressing the type of digital tool and the main PA outcomes (Multimedia Appendix 1). In detail, the type of digital tools searched included apps, wearables, social media, messaging, exergames, digital assistants, and different devices such as pedometers or accelerometers (Multimedia Appendix 1). No language restrictions were applied to the search strategy. Filters used were the range publication year from 2018 to 2025, the study design being an SR or meta-analysis, and the age range between 6 and 65 years. With regard to age, a comprehensive search for the age ranges 6‐65 years was performed first, as this was the target of the authors’ pilot study within the DARE Project, and afterward, studies targeting only children and adolescents (6‐17 y) were selected for the purposes of this study. The decision to conduct separate analyses for younger populations and adults was motivated by important age-related differences in the use of digital technologies. Young people tend to engage with digital tools that are distinct from those commonly used by adults. Moreover, these tools may influence behaviors in different ways across age groups. In particular, the impact of digital technologies on PA practice may vary according to developmental stage. For these reasons, age-specific analyses were considered necessary to ensure a more accurate interpretation of the findings.

References were managed and duplicates removed using Zotero (version 6.0.37, Corporation for Digital Scholarship). The selection process, with a first screening of titles and abstracts and a second screening of full texts, was conducted by 2 independent reviewers. Possible divergences were solved by discussion between the two reviewers or with the intervention of a third reviewer.

### Eligibility Criteria

The PICOS (Population, Intervention, Comparison, Outcome, and Study Type) criteria were used to derive the eligibility of the studies.

For the population*,* we selected healthy human participants in the age range of 6‐17 years. Healthy participants are those who are not diagnosed with any disease or who do not have disabilities that could affect PA outcome assessment. Reviews targeted to overweight or obese participants without another declared disease were retained. Moreover, populations aged 6‐65 years were initially included in the review, based on the needs of the project; afterward, studies targeting only populations aged between 6 and 17 years were selected for the present review.

For intervention, we had interventions using digital approaches to increase PA. Such digital approaches should have included the use of one or a combination of the following: IT devices (eg, smartphone, tablet, and PC); wearable activity trackers (eg, accelerometers and pedometers); applications (eg, stand-alone apps or apps associated with wearable devices); social media tools (eg, chatbots or conversational agents, and social networks); messaging tools (eg, email, SMS text messages, and podcasts); web-based tools (eg, websites, and video chats or video sharing); exergames, active video games, or gamification approaches; and synchronous or nonsynchronous PA coaching or training.

For comparison, we use any other intervention or no intervention.

For the outcome*,* we used PA as the primary or secondary outcome.

For study type, we used SRs and meta-analyses including RCTs. The following reviews were excluded: reviews of reviews or meta-analyses; scoping or narrative reviews; those not reporting PA-specific outcomes (eg, those reporting only SBs, only dietary or nutrition outcomes, or only weight-related outcomes); those focusing only on wearables that cannot be applied to huge populations for economical or organizational reasons (eg, virtual reality); those focusing on individuals (telemedicine); studies where the intervention was not delivered by at least 1 digital tool; papers where the digital component was used only to “measure” PA and not as a means to increase PA; those reporting only nondigital self-reported tools (such as paper questionnaires) for the assessment of PA; interventions for secondary or tertiary prevention (eg, targeted to individuals with preclinical signs); studies targeted to specific groups, such as smokers or pregnant women.

### Data Extraction

The data from the selected reviews were extracted by the same 2 independent reviewers that conducted the selection process, and the following information was reported: first author and year of publication; document type; design of studies included in the review; country; age group; special population; date range of the search; number of databases searched; digital device for intervention delivery; digital component for intervention delivery; focus of intervention or digital tool; outcomes; number of total studies included in the review; number of total RCTs in all reviews; number of RCTs of interest; and inclusion criteria, first author, and publication date of RCTs describing tools of interest.

For the primary studies identified within each review, the following information was extracted: first author and year of publication; country; age group; special population; school attendance; sample dimension and type; intervention duration, arms, or name; setting; focus task; theoretical foundation; digital component or components of intervention; nondigital component or components; digital device for intervention delivery; intervention group (IG) and control group (CG); device-based or self-reported PA measure; PA objective measurement tool; fitness tests assessed; other assessed measures; and effectiveness on PA or SB outcomes. Regarding outcome effectiveness, an intervention was classified as partially effective when positive effects were observed for some, but not all, PA outcomes.

### Data Synthesis Methods

Data regarding the characteristics of the different reviews and of the RCTs selected are presented as numbers and percentage frequencies. Effectiveness data were stratified by the type of device-based PA outcome and by the characteristics of the interventions. Differences between groups in relation to their effectiveness have been estimated through the Pearson chi-square test. All statistical estimates were conducted using STATA MP (version 12.1, StataCorp, 2011) software. As a meta-analysis was not feasible given the design of this review, heterogeneity between studies was explored using qualitative and descriptive methods combined with narrative synthesis. Sensitivity analyses were qualitatively performed as well to understand differences in populations, interventions, and outcomes that may explain variability in findings. Considered factors for sensitivity analyses were the quality of included reviews and RCTs, the study population characteristics, the intervention features, and the outcome measurement.

### Degree of Overlap

The extent of overlap of RCTs across the included SRs and meta-analyses was examined using the corrected covered area (CCA) approach [20]. As STATA does not provide a dedicated command for computing the CCA, a citation matrix was constructed, with primary studies listed in rows and the different SRs or meta-analyses in columns. A custom STATA script was developed to automate the calculation according to the formula: CCA = (N − r) / (*r*×c − r), where *N* represents the total number of publications, *r* the number of unique studies, and *c* the number of reviews or meta-analyses. The resulting values were converted to percentages by multiplying by 100. A CCA of 0% indicates no overlap (each review or meta-analysis includes only unique RCT); 0%‐5% reflects slight overlap; 6%‐10% moderate overlap; 11%‐15% high overlap; and values above 15% indicate very high overlap. A value of 100% denotes complete overlap, meaning that all reviews or meta-analyses include the same primary studies.

### Quality Assessment of the Reviews or Meta-Analyses

The methodological quality of included reviews and meta-analyses reporting randomized controlled interventions was assessed using the AMSTAR 2 (A Measurement Tool to Assess Systematic Reviews) tool [21]. This tool allows a quality assessment based on 7 critical and 9 noncritical domains, rather than a total score, generating an overall rating of “high” quality (those reviews or meta-analyses that meet 7/7 critical domains), “moderate” (6/7), “low” (those meeting all but one critical domain and missing few noncritical domains), and “critically low” (not meeting multiple critical domains).

### Quality Assessment of the RCTs

The methodological quality of each included RCT was evaluated using the 5 domains of the revised Cochrane Risk of Bias 2 (RoB 2) tool for RCTs [22]. Overall judgments for individual studies were derived according to the RoB 2 algorithm, categorizing studies as having a low risk of bias, some concerns, or a high risk of bias. A total of 2 reviewers conducted the assessments independently, and any discrepancies were resolved through consultation with a third reviewer.

## Results

### Search Flow Results

**Figure 1.**
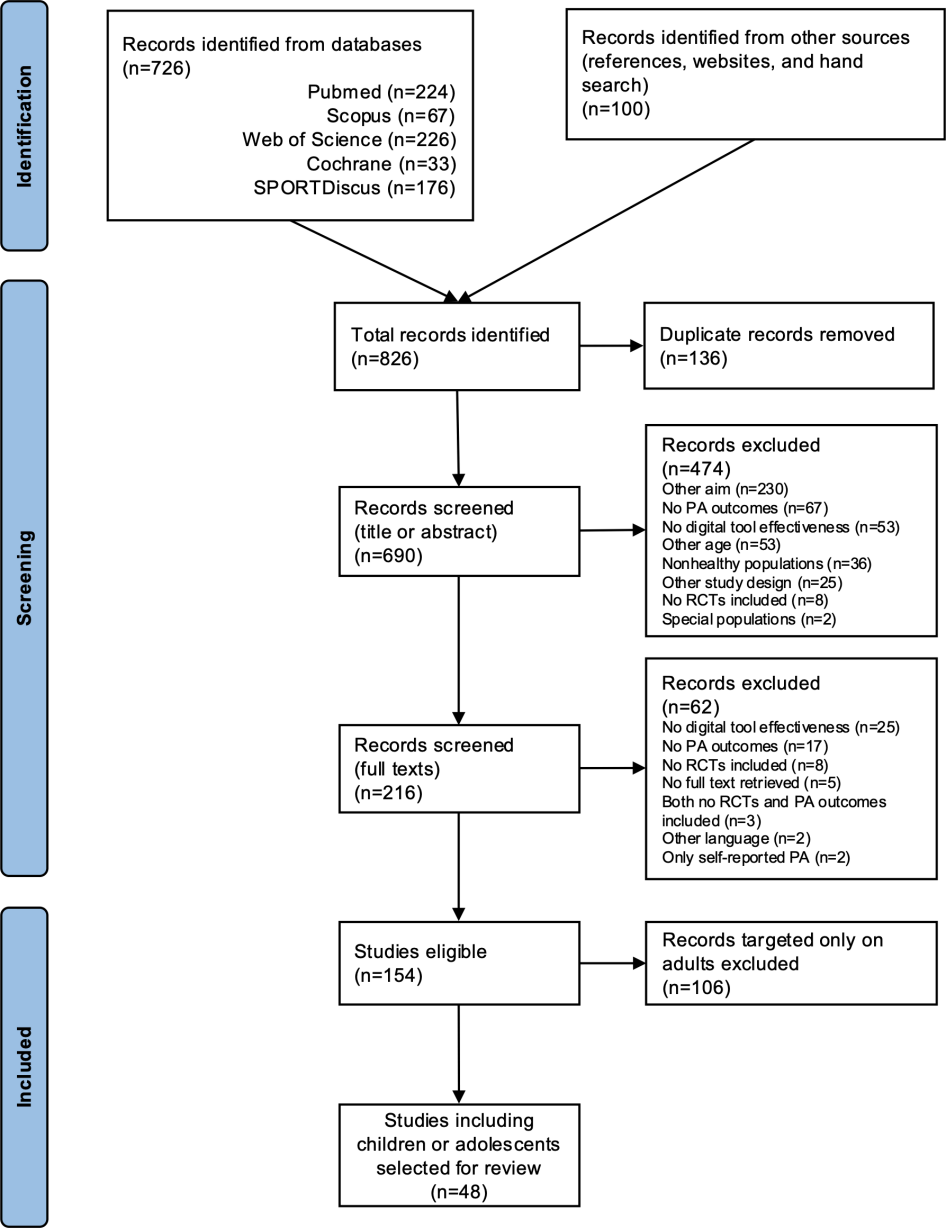
PRISMA flow diagram of the search. PA: physical activity; PRISMA: Preferred Reporting Items for Systematic Review and Meta-Analyses; RCT: randomized controlled trial.

Figure 1 shows the flow diagram of the search.

The database search included a total of 726 records, which were added to the 100 records identified by the manual search. After excluding 136 duplicates, a total of 690 reviews were screened by title and abstract, and subsequently, the full texts of the selected 216 records were examined. A total of 154 studies were eligible, but they included all-age populations. A further selection finally included a total of 48 SRs and meta-analyses targeted specifically to children and adolescents [23-70].

### Description of the SRs or Meta-Analyses

#### Characteristics of the SRs or Meta-Analyses

The characteristics of the included SRs or meta-analyses are shown in Multimedia Appendix 2, while Figure S1 Multimedia Appendix 3 provides a summary of the percentage frequencies of the different characteristics.

As shown in Figure S1 Multimedia Appendix 3, in 43.8% of the reviews, a meta-analysis was added to the SR [23,26,27,31,32,40,42,43,46,47,50,52,54,58,60,61,63,66-69]. The considered SRs or meta-analyses included any kind of study, both experimental and observational. The major part of SRs or meta-analyses was published by European research groups, mainly from the United Kingdom [28,29,32,34,39,41,45,59,61], Germany [23,24,33,47,50], Italy [25,38,55], and Spain [26,43,63]. The other European SRs or meta-analyses were published by research groups from Portugal [35,56], France [40], the Netherlands [44], and Denmark [65]. Less than one-third of the reviews were from research groups based in different Asian countries, such as China [31,49,53,54,60,66-69], Hong Kong [52], Korea [52], Malaysia [58,62], Iran [36], Singapore [37], and Qatar [41]. Most American SRs or meta-analyses were from the United States [30,47,48,57,71-75], and the others from Canada [51] and Brazil [42,64]. Only 2 reviews were published by Australian research groups [27,70].

Most reviews included studies using a miscellaneous assortment of device types [24,27,29,32-35,37,38,40-42,45,48,53,56,57,59,61,63,68-71] including mobiles, computers, tablets, and wearables, or mobiles and wearables, followed by only mobiles or smartphones [23,25,30,31,36,39,44,46,47,49-51,55,66] or only wearables [26,28,43,52,54,58,67], or consoles as devices for their gamification tools [60,62,64,65].

Parallelly, most SRs or meta-analyses searched for a miscellaneous assortment of different components for the intervention delivery [23,24,27,29-34,37,38,41,46,55-59,61,63,70], while a lower number searched only for gamification tools, including exergames, serious games, or games in devices [35,40,42,45,48,49,60,62,64,65,68,71], and in one case the exergame use was associated with coaching or telehealth [45]. Some reviews examined apps [25,36,44,47,50,51,66], and some investigated wearables [26,28,43,52,54,67]; 2 reviews were focused on chatbots [53,69] and only 1 on text messaging [39].

The focus task comprised exclusively of PAs in less than half of the reviews [24,28,30,31,33,37,40,41,44,45,47-50,52,54,57,58,60,61,63,76]; in the other reviews, the main outcomes were PAs associated with SB [23,26,38,39,43,67-69], with weight, obesity, or body composition [29,42,64,65], with diet [35] or physical fitness [66]; other reviews were focused on weight or obesity management [25,32,34,36,62,70], and on multiple health behaviors such as alcohol consumption, tobacco smoking, or diet and sleep, beyond PA and SB [27,46,51,53,55,56,59,71].

The selected reviews included an average of 19 (SD 12.2) experimental studies each for a total of 1023 studies. An average of 3.7 (SD 2.9) RCTs was present in each review. A total of 165 primary RCTs were identified across the 48 SRs or meta-analyses, according to the inclusion criteria (RCTs, healthy populations, PA outcomes present, ages between 6 and 17 years, published in the last 15 years, and effectiveness of digital tools assessed). The resulting CCA was 3.5%, indicating a slight degree of overlap. The 62 unique component studies identified were included in the final analysis [77-138].

#### Quality of the SRs or Meta-Analyses

The schematic results of the SRs’ and meta-analyses’ quality are shown in Multimedia Appendix 4. A high percentage of the reviews was rated as critically low (43.8%) [24-30,33-39,41,44,45,48,49,51,70], a rating that was often attributed to authors’ failure to address one of the critical criteria, namely providing a list of excluded studies along with justifications for their exclusion.

### Description of RCTs

#### General Characteristics

All extracted data from RCTs are shown in Multimedia Appendix 5. A total of 22 interventions did not have a specific name. The same intervention was described in 2 papers as follows: Active Teen Leaders Avoiding Screen-Time obesity prevention program [110,128], Dads and Daughters Exercising and Empowered intervention [115,116], Diab and Nano study [80,82], Girls on the Move intervention [120,124], MyMovez project [135,136], and the Raising Awareness of PA study [123,137]. The Nutrition and Enjoyable Activity for Teen Girls intervention was described in 3 papers [88,89,109].

Considerable heterogeneity was found for the sample dimension that was included between a minimum of 10 and a maximum of 1519 schoolchildren, and for intervention duration, which ranged between 2 weeks and 24 months.

In Figure S2 Multimedia Appendix 3, a summary of the main characteristics of the interventions, divided by general RCT aspects, is presented. The included RCTs were published between 2010 and 2025, mostly in America, and were primarily conducted in school settings, with others in home or community environments. Most interventions targeted middle school children, followed by primary school children and adolescents, with some focusing on overweight or obese students, and 11.3% conducted in low-income areas. The majority used a 2-arm design with IGs and CGs and mainly focused on PA alone, followed by PA combined with other behaviors or obesity-related outcomes.

A total of 38 interventions were based on different theories for behavioral changes. The most used theoretical models were the following: the self-determination theory by Deci and Ryan [139] and Ryan and Deci [140] was used in 16 RCTs [78,81,95-97,99,100,120,121,124,127,128,132,135,136,138]; the social cognitive theory by Bandura [141] was used in 15 RCTs [86,88,89,101,102,108,114,121,123,125,128,130,131,134,137]; 5 interventions [94,102,122,135,136] were based on the theory of planned behavior by Aizen [142]; the transtheoretical model of behavior change that was suggested by Prochaska and Velicer [143] was applied in 2 interventions [86,107].

#### Digital Aspects

Figure S3 Multimedia Appendix 3 shows a summary of the main characteristics of the trials related to the digital aspects of the intervention delivery. Most interventions included a wearable as a digital component of the intervention; in some cases, the wearable was a unique, tested digital tool [77,79,92,103,105,106,108,113,114,117,118,129], in some other trials, it was included in a multicomponent intervention [84,86,88,89,99,100,102,107,109,110,114-116,126,128,132,135-137]. Several RCTs used gamification, apps, web-based technologies, and text messaging; few interventions used social media, live coaching, or intelligent personal assistants. The most used digital devices were mobile phones or smartphones or wearables; 19.4% of interventions used the console or arcade machine and the computer, laptop, or tablet.

Around half of the RCTs used nondigital components for the intervention delivery, such as goals, rewards, incentives, advice, diaries, school programs, face-to-face educational sessions, lessons, or booklets (Multimedia Appendix 5).

A total of 53.2% of interventions used device-based measures only to evaluate PA outcomes, while the rest used device-based measures together with self-report measures, such as data from questionnaires or step diaries (Figure S3 Multimedia Appendix 3).

Device-based measurement tools were mainly accelerometers or activity trackers, that is, ActiGraph (Ametris, LLC) [80-83,85,86,88-90,92,93,98,99,101,102,104,109-112,117,120,123,124,128,130-133,137], Fitbit Flex or Fitbit Zip (Fitbit, Inc) [121,123,131,134-136], GENEActiv (ActivInsights Ltd) accelerometers [78,84,87] and Polar Active (Polar Products Inc) sensors [103,107,126,144]. Around one-third of the interventions used pedometers for step count, mainly Yamax (Polygon Direct [UK] Ltd) [77,79,94,108,113-116,129], Tractivity (Tractivity Ltd) [95-97], and Omron (OMRON Healthcare Co, Ltd) [106,118]. In 5 RCTs, other device-based measurement tools were embedded, for example, in the game machines such as the PlayStation (Sony Interactive Entertainment LLC) memory cards [82,93,111,112,122].

Device-based measures consisted mostly of moderate-to-vigorous physical activity (MVPA) or other PA outcomes such as vigorous physical activity (VPA), moderate PA, light physical activity (LPA), and total PA [80-82,85-88,88,92,93,95-98,101,103,104,109-112,117,119,121,122,126-128,130,131,133,137]; step counts were evaluated in half of the interventions [77,79,84,91,92,94-99,102,106,108,113-116,118,121,123,125,127,129-132,134-136,138].

A total of 19 RCTs also assessed physical fitness abilities through fitness tests; for example, cardiorespiratory fitness through the 20 m shuttle test [84,104,111,120], the 1-mile walk or run test [90,113], or the grip strength through the dynamometer [84,103,107,128]. A total of 86.9% of the interventions collected other outcomes, including anthropometry or body composition, cognitive or psychosocial aspects, dietary outcomes, clinical biomarkers, smoking, alcohol, sleep, and media use.

#### Effectiveness

As reported in Table 1 and Multimedia Appendix 5, a total of 54.8% of the interventions were not effective on the considered device-based PA outcomes [78,80-82,85,88-90,92,93,98,99,102,108-113,120-126,128-130,134-138]. The other RCTs showed effectiveness (37.1%) [77,79,83,84,86,94-97,100,101,104-107,114-116,118,127,131-133] or partial effectiveness (8.1%) [87,91,103,117,119] on one or more PA outcomes, meaning that the digital intervention had positive effects on some PA outcomes and not on other PA outcomes; for example, in the intervention of Comeras-Chueca et al [87], the Active video game increased the LPA, but not moderate PA, VPA, or total PA; or in the RCT from Petrušič et al [119], the digital placebo video games contributed to increasing the level of LPA but not of the VPA.

**Table 1.** Frequencies (in number and percentage) of the RCTs'^a^ effectiveness overall and by specific device-based outcome.^b^

Device-based outcomes	Total		Effective		Ineffective	
Total RCTs, n (%)	62 (100)		28^c^ (45.2)		34 (54.8)	
Total outcomes, n (%)	122 (100)		40 (32.8)		82 (67.2)	
Steps, n (%)	31 (50)		16 (25.8)		15 (24.2)	
MVPA^d^, n (%)	38 (61.3)		12 (19.4)		26 (41.9)	
LPA^e^, n (%)	13 (21)		4 (6.5)		9 (14.5)	
MPA^f^, n (%)	6 (9.7)		1 (1.6)		5 (8.1)	
VPA^g^, n (%)	9 (14.5)		1 (1.6)		8 (12.9)	
TPA^h^, n (%)	7 (11.3)		1 (1.6)		6 (9.7)	
LVPA^i^, n (%)	1 (1.6)		0 (0)		1 (1.6)	
CPM^j^, n (%)	12 (19.4)		2 (3.2)		10 (16.1)	
Active minutes, n (%)	5 (8.1)		3 (4.8)		2 (3.2)	

a RCT: randomized controlled trial.

b Referred to the 62 randomized controlled trials.

c A total of 5 of 28 interventions were partially effective, that is, effectiveness was demonstrated for some physical activity outcomes of the intervention.

d MVPA: moderate-to-vigorous physical activity.

e LPA: light physical activity.

f MPA: moderate physical activity.

g VPA: vigorous physical activity.

h TPA: total physical activity.

i LVPA: light-to-vigorous physical activity.

j CPM: counts per minute.

Moreover, some interventions demonstrated stronger effects when implemented over shorter durations or when assessed at short-term follow-up, typically within 3 to 6 months [91,103,118].

When stratifying RCTs’ effectiveness by the type of device-based outcomes, a total of 25.8% of the RCTs were effective in increasing the number of steps, while a similar percentage demonstrated no effectiveness (Table 1). The major part of the RCTs assessing MVPA was not effective in this increase; all the other device-based PA outcomes mostly did not increase after the digital intervention (Table 1). Totally, 67.2% of the PA outcomes were not positively affected by the intervention.

Stratified analyses of intervention characteristics (Table 2) showed higher proportions of effective interventions in trials conducted in Asian populations; those targeting PA as the sole primary outcome; interventions using wearables as the digital component or delivery device; trials measuring PA with pedometers; and interventions using specific devices (Tractivity, Omron, Polar Active, and Yamax) rather than ActiGraph or other accelerometers.

**Table 2.** Frequencies (in numbers and percentages) of RCTs’^a^ effectiveness on the device-based outcomes, by their characteristics.

Group	Total participants (N=62), n	Effective or partially effective on device-based PA^b^ outcomes		Ineffective on device-based PA outcomes		*P* value^c^
		Participants, n/N, (%)	Participants wrt^d^ the total cohort (ie, n/62×100), %	Participants, n/N, (%)	Participants wrt the total cohort (ie, n/62×100), %	
Age (years)						.26
Younger people (6-13)	39	20/39 (51.3)	32.3	19/39 (48.7)	30.6	
Older people (14-17)	21	8/21 (38.1)	12.9	13/21 (61.9)	21	
Younger people and older people (6-17)	2	0/2 (0)	0	2/2 (100)	3.2	
Continent						.06
America	24	10/24 (41.7)	16.1	14/24 (58.3)	22.6	
Asia	5	5/5 (100)	8.1	0/5 (0)	0	
Europe	17	8/17 (47.1)	12.9	9/17 (52.9)	14.5	
Oceania	16	5/16 (31.3)	8.1	11/16 (68.8)	17.7	
Setting						.09
School or other community	42	22/42 (52.4)	35.5	20/42 (47.6)	32.3	
Home	19	5/19 (26.3)	8.1	14/19 (73.7)	22.6	
Any setting	1	1/1 (100)	1.6	0/1 (0)	0	
Publication date						.92
2010‐2017	35	16/35 (45.7)	25.8	19/35 (54.3)	30.6	
2018‐2025	27	12/27 (44.4)	19.4	15/27 (55.6)	24.2	
Special populations						
Weight status						.20
Normal weight	48	25/48 (52.1)	40.3	23/48 (47.9)	37.1	
At risk of obesity	2	0/2 (0)	0	2/2 (100)	3.2	
Normal, overweight, or obese	3	1/3 (33.3)	1.6	2/3 (66.7)	3.2	
Overweight or obese	9	2/9 (22.2)	3.2	7/9 (77.8)	11.3	
Income						.01
Any income	55	28/55 (50.9)	45.2	27/55 (49.1)	43.5	
Low income	7	0/7 (0)	0	7/7 (100)	11.3	
PA level						.06
Sufficiently active	58	28/58 (48.3)	45.2	30/58 (51.7)	48.4	
Insufficiently active	4	0/4 (0)	0	4/4 (100)	6.5	
Intervention duration						.16
3 months or less	36	19/36 (52.8)	30.6	17/36 (47.2)	27.4	
More than 3 months	21	6/21 (28.6)	9.7	15/21 (71.4)	24.2	
N/A^e^	5	3/5 (60)	4.8	2/5 (40)	3.2	
Intervention arms						.54
2-arm	50	23/50 (46)	37.1	27/50 (54)	43.5	
3-arm	10	4/10 (40)	6.5	6/10 (60)	9.7	
4-arm	1	1/1 (100)	1.6	0/1 (0)	0	
N/A	1	0/1 (0)	0	1/1 (100)	1.6	
Theoretical foundation						.16
Yes	37	14/37 (37.8)	22.6	23/37 (62.2)	37.1	
N/A	25	14/25 (56)	22.6	11/25 (44)	17.7	
Focus task						.09
PA only	32	19/32 (59.4)	30.6	13/32 (40.6)	21	
PA and other	17	4/17 (23.5)	6.5	13/17 (76.5)	21	
Obesity, weight, body composition, or energy balance	10	3/10 (30)	4.8	7/10 (70)	11.3	
Other (health or sleep)	3	2/3 (66.7)	3.2	1/3 (33.3)	1.6	
Digital component						.68
App	3	1/3 (33.3)	1.6	2/3 (66.7)	3.2	
Gamification	18	8/18 (44.4)	12.9	10/18 (55.6)	16.1	
Miscellaneous	23	9/23 (39.1)	14.5	14/23 (60.9)	22.6	
Text messaging	2	1/2 (50)	1.6	1/2 (50)	1.6	
Wearable	11	7/11 (63.6)	11.3	4/11 (36.4)	6.5	
Web-based	4	2/4 (50)	3.2	2/4 (50)	3.2	
Nondigital component added						.42
Yes	32	16/32 (50)	25.8	16/32 (50)	25.8	
No	30	12/30 (40)	19.4	18/30 (60)	29	
Digital device for intervention delivery						.37
Computer, laptop, tablet, or iPod touch (Apple Inc)	8	4/8 (50)	6.5	4/8 (50)	6.5	
Console or arcade	12	5/12 (41.7)	8.1	7/12 (58.3)	11.3	
Mobile phone or smartphone	14	7/14 (50)	11.3	7/14 (50)	11.3	
Miscellaneous	10	3/10 (30)	4.8	7/10 (70)	11.3	
Wearable	12	8/12 (66.7)	12.9	4/12 (33.3)	6.5	
N/A	6	1/6 (16.7)	1.6	5/6 (83.3)	8.1	
Device-based and self-report measure						.68
Device-based only	33	17/33 (51.5)	27.4	16/33 (48.5)	25.8	
Device-based and self-reported	29	11/29 (37.9)	17.7	18/29 (62.1)	29	
Device for outcome measurement						.003
Accelerometer only	36	14/36 (38.9)	22.6	22/36 (61.1)	35.5	
Accelerometer and calorimeter	1	1/1 (100)	1.6	0/1 (0)	0	
Accelerometer and exergame in-built device	4	0/4 (0)	0	4/4 (100)	6.5	
Accelerometer and pedometer	4	0/4 (0)	0	4/4 (100)	6.5	
Pedometer only	16	13/16 (81.3)	21	3/16 (18.8)	4.8	
Exergame in-built device	1	0/1 (0)	0	1/1 (100)	1.6	
Brand of device for outcome measurement						.09
ActiGraph accelerometer	26	8/26 (30.8)	12.9	18/26 (69.2)	29	
Polar Active accelerometer	3	2/3 (66.7)	3.2	1/3 (33.3)	1.6	
New Lifestyles (NEW LIFESTYLES, Inc) accelerometer	2	1/2 (50)	1.6	1/2 (50)	1.6	
Fitbit Flex accelerometer	4	0/4 (0)	0	4/4 (100)	6.5	
Tractivity accelerometer	3	3/3 (100)	4.8	0/3 (0)	0	
GENEActiv accelerometer	2	1/2 (50)	1.6	1/2 (50)	1.6	
Yamax pedometer	9	6/9 (66.7)	9.7	3/9 (33.3)	4.8	
Omron pedometer	2	2/2 (100)	3.2	0/2 (0)	0	
Two different devices^f^	4	1/4 (25)	1.6	3/4 (75)	4.8	
Other^g^	7	4/7 (57.1)	6.5	3/7 (42.9)	4.8	
Device-based PA outcome						.21
Steps	31	16/31 (51.6)	25.8	15/31 (48.4)	24.2	
MVPA^h^	38	12/38 (31.6)	19.4	26/38 (68.4)	41.9	
Other PA outcomes^i^	31	11/31 (35.5)	17.7	20/31 (64.5)	32.3	
Risk of bias						.25
Low	25	12/25 (48)	19.4	13/25 (52)	21	
Some concern	30	16/30 (53.3)	25.8	14/30 (46.7)	22.6	
High	7	6/7 (85.7)	9.7	1/7 (14.3)	1.6	

a RCT: randomized controlled trial.

b PA: physical activity.

c Difference between groups estimated through the Pearson chi-square test.

d wrt: with respect to.

e N/A: not applicable.

f GENEActiv accelerometer and Misfit Ray (Fossil Group) accelerometer, ActiGraph accelerometer and New Lifestyles accelerometer (New Lifestyles, Inc), ActiGraph accelerometer and Sony Playstation 2 memory cards, and ActiGraph accelerometer and Omron pedometer.

g Walk4Life (Active Together) pedometer, Zamzee accelerometer (Firefly Design LLC), accelerometer (brand not available), MOX (Maastricht Instruments BV) accelerometer, Hoggan Health (Hoggan Scientific, LLC) exergame in-built device, Samsung mobile app Health pedometer (Samsung Electronics Co, Ltd), and movisens (movisens GmbH) accelerometer.

h MVPA: moderate-to-vigorous physical activity.

i CPM: counts per minute; LPA: light physical activity; LVPA: light-to-vigorous physical activity; VPA: vigorous physical activity; active minutes.

Examining in detail, the results on the digital components for intervention delivery, the gamification tools that were efficacious or partially efficacious on the device-based PA outcomes were the MobileKids Monster Manor installed on iPhone (Apple Inc); Xbox 360 (Microsoft Corp); games of the Nintendo Wii, such as Wii Sports-Tennis, Sonic and Mario at the Olympics or Celebrity Sports Showdown-Horse Racing, Just Dance 3; games from Kinect (Microsoft Corp), such as Kinect Your Shape: Fitness Evolved 2012, Disneyland Adventures and Kinect Sports Season 2, and Kinetic Adventures! Some games from Xbox 360 were efficacious in 2 interventions [104,131], and nonefficacious in 1 intervention from Staiano et al [130]. The game Dance Dance Revolution was efficacious when it was used in the intervention from Maloney et al [112], while in the intervention from Errickson et al [93], it did not show any effect on PA outcomes. The ineffective games were Diab and Nano, as well as active video games such as PlayStation or Sports and Dance Factory, Sport Champions, Move Fitness, Start the Party and Medieval Moves, Dance Star Party and Sorcery, Camp Conquer, a FitQuest smartphone exergame, and Dance Central.

Effective apps in increasing schoolchildren’s PA were the Dads and Daughters Exercising and Empowered app [115] and the mobile app MapMyFitness [127]; they both were developed quite recently and targeted to primary and secondary schoolchildren, respectively. The smartphone app “Eat Wisely and Move Happily” used in the Diet, Exercise and Cardiovascular Health–Children [145] was partially effective, while 3 apps were ineffective in increasing PA, such as the immersive app “Zombies Run! 5K training” and the nonimmersive app “Get Running-Couch to 5K” from the AIMFIT (Apps for Improving Fitness) [90], the app from the Active Teen Leaders Avoiding Screen-Time obesity prevention program [110], and from MyMovez project [135,136].

Conversely, higher proportions of ineffective interventions were observed in RCTs conducted in Australia or New Zealand; home-based trials; interventions targeting individuals at risk for obesity, overweight or obese participants, or mixed populations including overweight youth; and trials focused on low-income or insufficiently active children and adolescents. Interventions lasting longer than 3 months and those targeting PA alongside other behaviors or obesity-related outcomes were mostly ineffective (Table 2). App-based and mixed digital interventions also showed limited effectiveness. Trials using multiple delivery devices were largely ineffective, whereas combining accelerometers with exergame or pedometer devices, or using exergame-only devices, was associated with higher effectiveness.

Nearly 70% of the interventions using ActiGraph accelerometers were not effective, all interventions using Fitbit Flex were ineffective, and ineffectiveness increased to 75% when 2 devices were used. Overall, 68.4% of interventions did not increase MVPA, and 64.5% did not improve other PA outcomes. Chi-square analyses showed significant associations for low-income populations (*P*=.01) and outcome measurement device (*P*=.003), with additional trends for country of origin, setting, baseline PA level, intervention focus, and sensor brand (Table 2).

#### Quality of the RCTs

A total of 11.3% of the RCTs showed a high risk of bias, while the 48.4% had some concern (Multimedia Appendix 6). One of the critical points regards the “deviations from the intended interventions” (33.9% of the interventions) and “measurement of the outcome” (19.4%).

## Discussion

### Principal Findings

The present umbrella review provides an overview of SRs and meta-analyses that collected RCTs aimed at evaluating the effectiveness of digital approaches to increase PA in healthy children and adolescents. It summarizes and critically evaluates the digital intervention components, as well as the possible determinants and methodological limitations of their effectiveness.

Slightly less than half of the interventions reported either total or partial effectiveness of the digital tools in increasing device-based PA outcomes. A wide variation in study designs, target age groups, intervention objectives, digital devices, and intervention components was identified across the reviews and trials.

Effectiveness over the population target of the present review appears to be influenced by multiple factors, including the focus task of the intervention, the type of digital component used to deliver the intervention and its delivery device, the device used for the objective measurement of PA, some specific population target, and other characteristics such as the geographic region, the intervention setting, and the device brand.

### Country

#### Overview

A first observation concerns the underrepresentation of certain countries in the implementation of the included trials, particularly when considered in relation to the population size of countries with advanced economies across continents. Most interventions were conducted in North America, predominantly in the United States; however, this concentration does not adequately reflect the size and diversity of the North American population, because when compared with the approximately 380 million inhabitants of the United States and Canada [146], the number of interventions remains relatively limited. Around one quarter of the included RCTs were conducted in Europe, mainly in the United Kingdom, while another quarter originated from Australia and New Zealand; the latter proportion appears comparatively high given the much smaller population size of these countries (approximately 31 million) compared with Europe (approximately 744 million) [146]. In contrast, Asian countries with advanced economies were notably underrepresented despite their large combined population (approximately 206 million) [146].

Moreover, the influence of the country on the effectiveness of digital tools is confirmed by the trend observed after the nonparametric association analyses. Several factors may plausibly explain why digital tools appear to be more effective in increasing PA among children in Asian populations. Higher levels of digital literacy and early exposure to technology in many Asian countries (especially in the economically advanced regions) may facilitate greater engagement with apps, wearables, and gamified platforms. In several Asian contexts, structured educational systems, strong acceptance of technology, and high parental support may enhance adherence and implementation fidelity. Additionally, school curricula may better integrate digital health tools into daily routines, increasing consistency of exposure. Urban environments, limited outdoor spaces, and academic pressures may further increase the appeal of digital interventions, helping to explain regional differences in their effectiveness for promoting PA in children. Future research should aim to address this imbalance by promoting RCTs in underrepresented regions and deeply investigating the differences in the effectiveness between the different countries. Expanding the geographical diversity of intervention settings would improve the generalizability of findings and provide a more comprehensive understanding of the effectiveness of digital interventions across different cultural and socioeconomic contexts.

#### Age and Special Population Groups

This review identified a tendency for RCTs to collect fewer data on high school students compared with those in primary and middle school, suggesting a need for increased research focused on this age group. Furthermore, specific population subgroups, such as students who are overweight or obese, or those from low socioeconomic backgrounds, were rarely included in the reviewed trials. This finding is consistent with the observations reported by Kracht et al [14]. All the interventions conducted to low-income populations were significantly ineffective, which suggests that it would be urgent to plan digital interventions that can positively affect PA also in children and adolescents from low-income families. Similar results were observed for subpopulations of insufficiently active children and adolescents, for whom all digital interventions did not result in effectiveness. Accordingly, future intervention studies should place greater emphasis on the inclusion of these underrepresented subgroups when designing and evaluating digital interventions.

#### Setting

With regard to the intervention setting, community-based environments, including schools, were substantially more prevalent than home-based settings, and a significant trend of difference in effectiveness for increasing PA was observed between these 2 contexts. As demonstrated in previous studies, social support, together with parental involvement and responsibility in implementing interventions with their children, plays a crucial role in facilitating behavior change [147,148]. Moreover, the ecological model of health and disease highlights how the onset of chronic conditions is influenced by environmental factors, beginning within the family context and extending to schools and the broader community [149]. Accordingly, both school and home environments are essential for the development of interventions aimed at modifying lifestyle behaviors [150,151]. Future research should therefore take greater consideration in conducting RCTs within home-based settings.

#### Focus Task

PA was a primary focus in most reviews, followed by the weight or obesity management tasks, and this was also reflected in the RCTs extracted by the reviews. Those interventions that had only PA as an outcome were apparently more effective than those focusing primarily on weight or obesity or PA together with other variables. Even though the explanation for this effect is not clear, it could be hypothesized that closer and more specific attention to a topic could improve the intervention procedure to then obtain more effective results. From a theoretical perspective, behavior change frameworks such as the self-determination theory [139] (which has been used in several RCTs of the present overview) suggest that interventions targeting clearly defined mechanisms (eg, motivation, capability, and opportunity) are more likely to be implemented with fidelity and evaluated appropriately. Narrowly focused interventions allow for clearer operationalization of outcomes and improved alignment between intervention components and measurement strategies. Empirically, previous SRs of digital PA interventions in children and adolescents have shown that interventions with clearly specified objectives and well-defined outcomes tend to report more consistent and interpretable effects compared with broadly defined or multicomponent interventions [66,152,153]. Moreover, methodological guidance for complex interventions (eg, the Medical Research Council framework [154]) emphasizes that precise intervention focus facilitates better study design, implementation, and evaluation.

#### Theoretical Foundation

This review revealed that studies including a theoretical foundation in the trial procedure seemed to be less effective than those that did not report any theory. This result is not in line with the literature reporting that the behavior change techniques and theoretical frameworks represent the core mechanisms through which interventions exert their effects. Although the most widely adopted standardized taxonomy of behavior change techniques was introduced only in 2013 [14], potentially constraining its use in earlier primary studies and reviews, the RCTs included in the present review that incorporated theoretical frameworks were predominantly conducted and published after this date. This advocates the need for reviewing and specifically addressing target populations with all the behavior change techniques and theories on the basis of digital tools interventions.

#### Digital Components for the Intervention Delivery

##### Gamification

The gamification was a digital component frequently investigated in the reviews and a commonly used tool in the retrieved RCTs. This is expected when we consider the target age of the populations under study; gamification tools such as exergames, serious games, or games embedded in devices are commonly used in this age as they are more appealing than other digital intervention tools. Gamification is intended as the use of suggestive digital games and game elements such as points or reward systems in nongame contexts, to influence users’ behavior and motivation [155]; gamification targeting PA is specifically designed to involve children in movement-based gameplay, encouraging them to be more active, as many common exergames (also called active video games) do. Recently, the concept of serious games has been of great interest, as they are designed for a primary purpose other than pure entertainment, thus having mainly an educational aim. However, there is often misleading information in the literature about the use of the term “serious game” because some authors define a serious game as any game used for an intervention study; that is, Liu et al [145] in their meta-analysis included different games that showed a significant effect of serious games on the increase of PA in children. In our work, there was no intervention reporting the term serious games, but only “exergames,” “role-playing videogames,” “active video games,” or “arcade machines,” which could be considered as serious games because they were designed or used for the purpose of the intervention. The term “serious game” should then be clarified in future publications to shed light on an agreement between the results of the different interventions.

In summary, it was expected that interventions using gamification and apps as digital components could be more effective than other tools in increasing the PA of young populations, but this was not found out in this umbrella review. The explanation is not so easy because different factors can influence the effectiveness of digital interventions, but it would be useful to explore in detail the single intervention, which is not the purpose of this review. Our recommendation, therefore, is that future studies should plan interventions using accurate gamification tools and apps that could be more effective for schoolchildren’s PA.

##### Wearables

The major part of interventions testing a wearable as a unique delivery tool and component reported effective results on increasing PA. These results are in line with the literature stating that wearable technologies have emerged as important digital tools for promoting PA among children and adolescents [156-158]. By providing real-time feedback, goal setting, and self-monitoring features, wearables can enhance awareness of movement behaviors and motivate users to increase daily activity levels. In younger populations, these devices support the development of self-regulation skills and reinforce positive behavior through immediate reinforcement and gamified elements. Moreover, wearable-based interventions can be integrated with mobile apps and social features, facilitating parental involvement, peer support, and structured activity challenges. Importantly, wearables also enable objective assessment of activity patterns, improving the accuracy of outcome measurement in intervention studies. Collectively, these features position wearable technologies as effective and scalable tools for supporting PA promotion in pediatric populations. It is suggested that these devices should be prioritized in future RCTs as intervention delivery tools.

##### Web-Based Tools, Apps, and Text Messages

Very few interventions examined the effects of web-based tools, mobile apps, and text messaging as unique components of the interventions, as these tools were generally included in a multicomponent intervention. Similar proportions of effective and ineffective outcomes between these 3 tools were observed in this review. In particular, RCTs that used web-based components were mainly addressed to older children attending middle or high schools, in contrast with gamification approaches that were targeted principally to schoolchildren from elementary schools, which is consistent with findings from the international literature [159-162]. Overall, these digital approaches demonstrate potential but yield inconsistent effects on PA promotion in schoolchildren [163].

### Nondigital Components

Nondigital components were used in slightly more than half of the interventions, and they were mostly goals, rewards, and incentives. Previous works have shown that multicomponent interventions, including nondigital components such as counseling for children and parents, class seminars, and health promotion curricula, could have a positive impact on PA outcomes [159]. Therefore, different communication channels should be combined with digital tools because they are based on the possible behavior change [164].

### Digital Device for Intervention Delivery and for PA Outcome Assessment

Most SRs or meta-analyses and RCTs included studies that used smartphones as devices for the intervention delivery, and this is consistent with the fact that they are the most common devices used to access the internet games, apps, or other digital components [165]. Effectiveness was not likely to be affected by the specific device. The same could be stated for the use of devices such as console or arcade machines, computers, tablets, or iPod touch in relation to the increase in PA outcomes. The use of a miscellaneous array of devices, instead, revealed a major proportion of ineffective interventions.

Other frequently used devices were the wearables, in most cases overlapping with the wearable that was used as a component of the digital intervention delivered. The main used wearables were accelerometers and pedometers, such as ActiGraph, Polar Active, or Tractivity, all tools that have been widely validated and broadly used [166-168]. Additionally, Yamax or Omron pedometers were mainly used for the step count measurement. The same type of device was used within the intervention for the assessment of PA outcomes. Greater significant effectiveness was found for the use of pedometers, compared for example to the accelerometers. Pedometers may be more effective than accelerometers in increasing PA among children and adolescents because they provide simple, easily interpretable feedback, such as step counts, that is readily understood by young users. This immediacy can enhance motivation and goal setting, particularly when daily step targets are used. Pedometers are also less complex and often more user-friendly, which may promote consistent wear and engagement. In contrast, accelerometers are typically designed primarily for measurement rather than behavior change and often provide limited or delayed feedback to participants [67,77,169]. The visibility of step counts can encourage self-monitoring and reinforce active behaviors throughout the day. As a result, pedometers may better support behavior change processes in youth populations.

### Device-Based PA Outcomes

Some considerations have to be made about the specific outcomes that were assessed through the devices. Step count was positively affected by the digital intervention in half of the considered interventions, while MVPA or other PA outcomes, such as light-to-vigorous PA, LPA, MVPA, VPA, counts per minute, and active minutes, did not increase in the majority of the interventions. The failure in increasing step or other PA outcomes could be explained by supposing that engagement with the technology declined over time, reducing sustained behavior change. Some interventions relied primarily on monitoring without incorporating sufficient behavior change techniques such as goal setting, feedback, or rewards.

Limited integration of social support from parents, peers, or schools may also have weakened intervention effects [170]. In addition, interventions of short duration may have been insufficient to produce measurable increases in habitual PA, or those that were too long may have led to a loss of interest [171]. Technical issues, poor usability, or lack of age-appropriate design could have further limited children’s and adolescents’ motivation to increase daily steps and other PA outcomes [44]. Finally, measurement challenges and high baseline MVPA levels in some samples may have limited the capacity to observe significant increases [172].

### Overlapping

The observed slight degree of overlap indicates that most SRs and meta-analyses include different primary studies. This statement supports more robust and balanced conclusions in this umbrella review, as the risk of double-counting evidence is reduced, and the independence of findings across reviews is strengthened. Although a slight overlap enhances the credibility of the overall synthesis by ensuring that conclusions are not driven by a small number of repeated trials, it may also increase heterogeneity, as findings are drawn from diverse interventions and populations [173]. Interpretation, therefore, requires careful consideration of contextual differences.

### Quality of the Studies

The critically low rating of the major part of the SRs and meta-analyses was frequently due to the omission of a key methodological requirement, specifically the reporting of excluded reviews and the reasons for their exclusion. The lack of transparency regarding excluded reviews could limit the completeness of the evidence base and the potential for selection bias.

With regard to the quality assessment of the RCTs, the domain of the RoB 2 tool “deviations from the intended interventions” was particularly critical. Digital interventions strongly depend on user engagement, adherence, and correct use of the technology, which are often inconsistent in younger populations. Children and adolescents may discontinue use, use the tools incorrectly, or engage at varying intensities. When participants deviate from the assigned intervention, the estimated intervention effect may be biased, especially if these deviations differ systematically between IG and CGs [174]. When not properly addressed analytically, they may lead to overestimation or underestimation of intervention effects or heterogeneity in reported outcomes.

### Limitations

A limitation of the present umbrella review is that several included SRs and meta-analyses were of critically low methodological quality; however, excluding all such reviews would have resulted in the omission of a substantial proportion of relevant evidence. This implies a potential and substantial influence on the interpretation of evidence of the digital tools’ effectiveness on increasing PA in children and adolescents. For example, methodological weaknesses, such as unclear inclusion criteria, incomplete search strategies, poor transparency in data extraction and synthesis, may reduce reproducibility and confidence in findings; inadequate assessment of risk of bias can result in overestimation or underestimation of intervention effects; insufficiently explored heterogeneity may mask important differences between intervention types or populations; inconsistent outcome definitions and measurement methods further limit comparability across reviews [175]. Therefore, the findings of this umbrella review should be interpreted with caution.

Similarly, RCTs assessed as having a high risk of bias constituted a substantial portion of the evidence base; therefore, excluding them would have been detrimental to the review. This could have influenced the results of this umbrella review, in the sense of overestimating the effectiveness of interventions, masking real differences between types of digital tools, such as mobile apps, wearable devices, or exergames. Common sources of bias, including inadequate randomization, lack of blinding, and high attrition, can systematically skew outcomes. Overall, the presence of high-risk trials weakens the ability to grade the certainty of evidence and may ultimately lead to inappropriate or poorly informed recommendations for practice and policy. Moreover, the lack of certainty of assessment (eg, through the GRADE [Grading of Recommendations, Assessment, Development, and Evaluation] tool) was a deliberate choice; it is a common and currently recognized practice in the field that a significant portion of published umbrella reviews do not assess the certainty of evidence, particularly when authors focus instead on the methodological quality of the included reviews using the AMSTAR 2 tool [176,177].

Another limit of this review is that it did not quantitatively synthesize through a meta-analytical approach (meta-meta-analyses or meta-analysis of the RCTs) the overall effectiveness, the heterogeneity, and the effects obtained after stratification and sensitivity analyses. Digital tools such as apps, wearables, exergames, and gamified platforms often differ in content, intensity, duration, and implementation context, making direct comparisons challenging. This variability limits the ability to synthesize findings quantitatively and reduces confidence in pooled effect estimates. In this overview, heterogeneity was explored through qualitative methods combined with narrative synthesis, and this approach helped in the identification of patterns and potential moderators even when quantitative pooling is not possible. Heterogeneity may obscure true intervention effects, as positive outcomes observed in some interventions may not be generalizable across different settings or age groups [178]. It also complicates the identification of which specific digital components or delivery strategies are most effective for schoolchildren. Consequently, conclusions must be framed more descriptively, and recommendations for practice and policy should emphasize context-specific effectiveness rather than universal applicability. Therefore, the authors envisage that the next step is to conduct a meta-analysis of RCTs to more robustly assess the effects of digital approaches on increases in PA. Such analyses should be complemented by sensitivity analyses, for example, excluding interventions of lower methodological quality, to enhance the robustness and interpretability of the findings.

As some of the included RCTs were conducted during the COVID-19 pandemic (2020‐2022) or in the postpandemic period, changes in PA patterns and digital tool use during these years may have influenced the observed outcomes [179], and this should be considered for the interpretation of results.

### Strengths

This umbrella review was based on rigorous and systematic methodology, relying on reviews that collected RCTs and that used objective device-based measures of PA. These measures improve the reliability of the review by providing more accurate and unbiased outcome assessment than self-reported data. Moreover, they reduce recall and social desirability bias, enhance comparability across studies, and allow more precise detection of changes in activity levels. As a result, they strengthen the quality of the evidence base, reduce heterogeneity related to outcome measurement, and increase confidence in conclusions regarding the effectiveness of digital interventions [180,181].

Risk of bias was evaluated at both the review and primary study levels, given that only a small subset of interventions from each review met the inclusion criteria for inclusion in this umbrella review. As a result, assessing risk of bias exclusively at the review level may have led to misleading conclusions, because the original reviews incorporated a broader evidence base.

Another strength of this review is its innovative character, as it is among the first umbrella reviews to comprehensively synthesize evidence from recent SRs and meta-analyses on digital PA interventions in children and adolescents. It provides novel comparative insights into methodological features and population subgroups that may drive or limit the success of digital approaches.

The findings of this work have several important real-world implications and contributions. They provide evidence-based guidance for practitioners, schools, and policymakers on which digital tools are more likely to effectively increase PA in children and adolescents, supporting more informed decision-making. The results highlight the importance of selecting appropriate devices, outcome measures, and intervention designs to maximize impact in real-world settings. This work also identifies gaps in equity and population coverage, informing future interventions aimed at reducing disparities in PA. Finally, the findings contribute to the optimization and scaling of digital health interventions by clarifying which approaches are most promising for practical implementation.

### Recommendations for Future Research and Implications

While the authors envisage quantitatively analyzing the effect of the interventions on the specific PA outcomes in children and adolescents in a future study through meta-analytic procedures, they wish to guide the strengthening of rigor for future SRs and interventions and support greater engagement in PA during childhood and adolescence.

First of all, the selection of device-based measures, which are able to provide reliable and comparable data, should be stressed. A clarification on the way to use gamification should be provided, together with a clearer explanation of the term “serious game” when it is used within the interventions. The importance of incorporating gamification tools, wearables, and/or apps as integral components of interventions should be highlighted, as they may be particularly effective for school-aged children [74,163].

Future interventions should place greater emphasis on conducting RCTs within home-based settings [150]. PA management should be the primary aim of the intervention; limiting the focus of the RCT to PA, without going too far into other outcomes that may cause a loss of the focus, could help in obtaining accurate data and effective outcomes [182]. There is a need to intensify RCTs targeted at high school populations, also considering that this age range is particularly engaged with digital technologies. Sustaining engagement over time remains a key challenge [183], highlighting the importance of developing and evaluating strategies that support long-term participation and adherence to digital interventions.

Better planning of interventions targeted to low-income and to insufficiently active subpopulations of children and adolescents represents a key aspect [184]. Future research should aim to promote and support RCTs in underrepresented regions, particularly in Asian and other non-Western high-income countries.

A greater emphasis on methodological transparency throughout the review and intervention selection process is suggested; in particular, clear and explicit criteria for study inclusion and exclusion should be reported. Transparency is needed in quality assessment procedures, including the rationale for quality scoring and study weighting; in addition, future reviews should clearly describe how methodological quality influences the interpretation of findings. The need for systematic evaluation and deliberate selection of behavior change techniques and theoretical models that are explicitly tailored to specific target populations within digital intervention contexts is underscored [185].

The results of this umbrella review can suggest practical actions both to schools and policymakers. Schools should avoid embedding PA as a minor component within broad well-being or educational programs; can integrate wearable-based programs (eg, step challenges and activity feedback) into physical education or daily routines; should favor pedometers or basic wearables over self-report tools to monitor student activity; and should accurately choose the type of device when implementing digital PA programs. Schools in disadvantaged areas may require additional support, such as teacher training, parental engagement strategies, or longer intervention duration.

Policymakers should prioritize funding and endorsement of interventions where PA improvement is explicitly defined, measured, and prioritized; can justify investments in wearable technologies as scalable and effective tools for youth PA promotion; can recommend objective PA monitoring standards in school-based programs to improve evaluation quality and accountability; can develop guidelines specifying preferred assessment tools to improve program effectiveness and comparability; should avoid one-size-fits-all approaches and allocate additional resources to adapt digital interventions to low-income contexts.

### Conclusions

This innovative study represents one of the first umbrella reviews describing digital PA interventions in youth, determinants of effectiveness, and methodological gaps. It provides evidence-based guidance for practitioners, schools, and policymakers on which features of the interventions are more likely to effectively increase PA in children and adolescents, supporting more informed decision-making. Interventions appear to be most effective when they prioritize focused PA objectives, incorporate wearables as both the core digital intervention component and the delivery device, use pedometers to measure PA outcomes, and choose accurately the device to assess PA as a quality criterion in program selection.

All these adaptations in future studies could be of great importance to generating high-quality primary RCTs and provide consistent conclusions about the usefulness of digital approaches to increase PA. The findings of this umbrella review have important implications for public health practice and policy. Digital PA interventions represent a scalable, cost-effective approach to addressing low PA levels among children and adolescents, a key modifiable risk factor for noncommunicable diseases across the life course. By identifying which types of digital tools show the greatest potential and which design and contextual factors influence effectiveness, this review supports more strategic investment in evidence-based digital solutions within schools, communities, and health care systems. The results also underscore the need for public health stakeholders to move beyond one-size-fits-all approaches and to ensure that digital interventions are developmentally appropriate, engaging, and equitably accessible, particularly for underserved populations. Strengthening methodological rigor in future research will improve the reliability of evidence used to guide policy decisions and large-scale implementation.

Collectively, these insights can help inform integrated public health strategies that leverage digital innovation to promote lifelong PA habits, reduce health inequalities among youth, and the burden of chronic diseases in adulthood.

## Supplementary material

10.2196/75769 Multimedia Appendix 1 Search strings.

10.2196/75769 Multimedia Appendix 2 Characteristics of the retrieved systematic reviews or meta-analyses.

10.2196/75769 Multimedia Appendix 3 Figures in percentage frequencies.

10.2196/75769 Multimedia Appendix 4 AMSTAR 2 quality rating table of the selected systematic reviews and meta-analyses. AMSTAR 2: A Measurement Tool to Assess Systematic Reviews.

10.2196/75769 Multimedia Appendix 5 Characteristics of the included randomized controlled trials.

10.2196/75769 Multimedia Appendix 6 Risk of bias of the selected randomized controlled trials.

10.2196/75769 Checklist 1 PRISMA checklist.

10.2196/75769 Checklist 2 PRIOR checklist.

## Abbreviations

AIMFIT: Apps for Improving Fitness
AMSTAR 2: A Measurement Tool to Assess Systematic Reviews
CCA: corrected covered area
CG: control group
DARE: Digital Lifelong Prevention
GRADE: Grading of Recommendations, Assessment, Development, and Evaluation
IG: intervention group
LPA: light physical activity
MVPA: moderate-to-vigorous physical activity
PA: physical activity
PICOS: Population, Intervention, Comparison, Outcome, and Study Type
PRIOR: Preferred Reporting Items for Overviews of Reviews
PRISMA-S: Preferred Reporting Items for Systematic Reviews and Meta-Analyses Literature Search Extension
PROSPERO: International Prospective Register of Systematic Reviews
RCT: randomized controlled trial
RoB 2: Risk of Bias 2
SB: sedentary behavior
SR: systematic review
VPA: vigorous physical activity
