# Supplementary material for: Digital Tools’ Effectiveness on Physical Activity Outcomes in Children and Adolescents: Umbrella Review
Source: JMIR Public Health Surveill. 2026 Mar 24;12:e75769. doi: 10.2196/75769 (PMC13013097; doi:10.2196/75769)
Supplement: Multimedia Appendix 1 — Search strings. [file publichealth-v12-e75769-s001.docx]

**PubMed**

*Filters: range publication year 01/01/2018 – 13/11/2025; age 6-65 years; systematic review, meta-analysis*

[Title/Abstract]

(digital OR digital tool OR eHealth OR mHealth OR app OR application OR mobile OR smartphone OR smartwatch OR tablet OR exergame* OR gamification OR active videogame* OR messaging OR SMS OR text messag* OR email OR social media OR social network OR video OR podcast OR wearable* OR activity tracker* OR activity monitor* OR accelerometer OR pedometer OR web coaching OR web train* OR virtual coach* OR chatbot OR digital assistant) AND (physical activity OR step OR MVPA) NOT (ill OR illness OR disorder* OR disease* OR disabilit* OR patient* OR clinic* OR survivor* OR surgery)

Total records: 224

**Scopus**

( TITLE-ABS-KEY ( digital OR digital tool OR eHealth OR mHealth OR app OR application OR mobile OR smartphone OR smartwatch OR tablet OR exergame OR gamification OR active videogame OR messaging OR SMS OR text message OR email OR social media OR social network OR video OR podcast OR wearable OR activity tracker OR activity monitor OR accelerometer OR pedometer OR web coaching OR web train OR virtual coach OR chatbot OR digital assistant ) AND TITLE-ABS-KEY ( physical activity OR step OR MVPA ) AND NOT TITLE-ABS-KEY ( ill OR illness OR disorder OR disease OR disabilities OR disability OR patient OR clinic OR survivor OR surgery ) AND NOT TITLE-ABS-KEY ( scoping review OR narrative review OR umbrella review OR protocol ) AND NOT TITLE-ABS-KEY ( preschool OR pre-school OR older OR elder OR aging ) ) AND ( LIMIT-TO ( DOCTYPE , "re" ) ) AND ( LIMIT-TO ( PUBYEAR , 2018 ) OR LIMIT-TO ( PUBYEAR , 2020 ) OR LIMIT-TO ( PUBYEAR , 2021 ) OR LIMIT-TO ( PUBYEAR , 2022 ) OR LIMIT-TO ( PUBYEAR , 2023 ) OR LIMIT-TO ( PUBYEAR , 2024 ) OR LIMIT-TO ( PUBYEAR , 2025 ) )

Total records: 67

**Web of science**

*Filters: range publication year 01/01/2018 – 13/11/2025; systematic review, meta-analysis*

[Abstract]

(digital OR digital tool OR eHealth OR mHealth OR app OR application OR mobile OR smartphone OR smartwatch OR tablet OR exergame OR gamification OR active videogame OR messaging OR SMS OR text message OR email OR social media OR social network OR video OR podcast OR wearable OR activity tracker OR activity monitor OR accelerometer OR pedometer OR web coaching OR web train OR virtual coach OR chatbot OR digital assistant) AND (physical activity OR step OR MVPA) NOT AB (ill OR illness OR disorder OR disease OR disabilities OR disability OR patient OR clinic OR survivor OR surgery) AND (systematic review OR meta-analysis) NOT (scoping review OR narrative review OR umbrella review OR protocol) NOT (preschool OR pre-school OR older OR elder OR aging)

Total records: 226

**Cochrane Database of Systematic Reviews**

[Title Abstract Keyword]

(digital OR digital tool OR eHealth OR mHealth OR app OR application OR mobile OR smartphone OR smartwatch OR tablet OR exergame OR gamification OR active videogame OR messaging OR SMS OR text message OR email OR social media OR social network OR video OR podcast OR wearable OR activity tracker OR activity monitor OR accelerometer OR pedometer OR web coaching OR web train OR virtual coach OR chatbot OR digital assistant) AND (physical activity OR step OR MVPA) NOT AB (ill OR illness OR disorder OR disease OR disabilities OR disability OR patient OR clinic OR survivor OR surgery) AND (systematic review OR meta-analysis) NOT (scoping review OR narrative review OR umbrella review OR protocol) NOT (preschool OR pre-school OR older OR elder OR aging)

Total records: 31

**SPORTdiscus/EBSCO**

*Filters: range publication year 01/01/2018 – 13/11/2025; age 6-65 years*

AB (digital OR digital tool OR eHealth OR mHealth OR app OR application OR mobile OR smartphone OR smartwatch OR tablet OR exergame OR gamification OR active videogame OR messaging OR SMS OR text message OR email OR social media OR social network OR video OR podcast OR wearable OR activity tracker OR activity monitor OR accelerometer OR pedometer OR web coaching OR web train OR virtual coach OR chatbot OR digital assistant) AND AB (physical activity OR step OR MVPA) NOT AB (ill OR illness OR disorder OR disease OR disabilities OR disability OR patient OR clinic OR survivor OR surgery) AND AB (systematic review OR meta-analysis)

NOT AB (scoping review OR narrative review OR umbrella review OR protocol) NOT AB (preschool OR pre-school OR older OR elder OR aging)

Total records: 176
