# Supplementary material for: Digital Tools’ Effectiveness on Physical Activity Outcomes in Children and Adolescents: Umbrella Review
Source: JMIR Public Health Surveill. 2026 Mar 24;12:e75769. doi: 10.2196/75769 (PMC13013097; doi:10.2196/75769)
Supplement: Multimedia Appendix 2 — Characteristics of the retrieved systematic reviews or meta-analyses. [file publichealth-v12-e75769-s002.docx]

**Table S1.** Characteristics of the retrieved systematic reviews/meta-analyses.

| **First author, publication year, country** | **Document type**  **Design of studies included in the review** | **Age group (age range) - special populations** | **Date range of the search** | **N. of databases searched** | **Digital component (device) for intervention delivery** | **Focus behavior of intervention/digital tool** | **N. of total studies included in the review** | **N. of RCTs of interest - inclusion criteria** | **First author and publication year of RCTs of interest** | **Quality assessment** |
| --- | --- | --- | --- | --- | --- | --- | --- | --- | --- | --- |
| Au WW, 2024, Hong Kong | SR and MA  Experimental (RCTs) | Children and adolescents | <Sep 2023 | 4 | Wearables (wearables) | PA | 21 | 9 - healthy, published >2009 | Baldursdottir, 2017 Guagliano, 2020 Lee, 2012 Lubans, 2011 Lubans, 2012 Morris, 2019 Stabelini Neto, 2016 Ruotsalainen, 2015 Smith, 2014 | Moderate |
| Baumann H, 2022, Germany | SR and MA  Experimental (RCTs) | Children and adolescents (0-21) | Jan 2000-Mar 2021 | 5 | Mix (mobiles) | PA and SB | 11 SR (10 MA) | 3 - age >6, healthy, PA outcomes, RCTs | Chen, 2019 Direito, 2015 Van Woudenberg, 2018 | Moderate |
| Böhm B, 2019, Germany | SR  Experimental (any), observational (cohort, cross-sectional) | Children and adolescents (6-18) | 2012-2018 | 5 | Mix (mobiles, wearables) | PA | 7 (5 - with tools of mHealth 2 - with wearable activity trackers) | 4 - PA outcomes | Dewar, 2014 Direito, 2015 Garde, 2016 Lubans, 2016 | Critically low |
| Bonvicini L, 2022, Italy | SR  Experimental (any), observational (cohort) | Children and adolescents (1–18), and parents | Jan 2014-Dec 2020 | 7 | Apps (smartphones) | Obesity | 19 | 1 - age >=6, PA outcomes, RCTs | Jake-Schoffman, 2018 | Critically low |
| Casado-Robles C, 2022, Spain | SR and MA  Experimental (any) | Children and adolescents (5-18) | <Dec 2020 | 8 | Wearables (wearables) | PA and SB | 45 SR (40 MA) | 6 - digital tools for delivery, published >2009, published in journals (no thesis), RCTs | Baldursdóttir, 2017 Guagliano, 2020 Jauho, 2015 Morris, 2019 Smith, 2014 Thompson, 2016 | Critically low |
| Chai LK, 2022, Australia/ Sweden | SR  Experimental (RCTs, controlled pre-post, controlled time series) | Children (5–12) | < Feb 2021 | 4 | Mix (mix) | Obesity | 23 | 4 - age >=6, PA outcomes, RCTs | Jake-Schoffman, 2018 Maddison, 2014 Morgan, 2019 Trost, 2014 | Critically low |
| Champion KE, 2019, Australia | SR and MA  Experimental (RCTs) | Children and adolescents (mean age 13.41, SD 1.52) | Jan 200-Mar 2019 | 4 | Mix - Internet, tele-health (mobiles, computers, tablets) | Multiple health behaviors (alcohol consumption, smoking, diet, PA, SB, sleep) | 22 SR (11 MA) | 1 - PA outcomes, published >2009 | Ezendam, 2012 | Critically low |
| Chen J, 2025, China | SR  Experimental (RCTs) | Children, adolescents, adults | Jan 2010-July 2024 | 12 | Chatbots (telephone, smart-speakers, websites, apps) | Multiple health behaviors (diet, PA, smoking) | 7 | 1 - PA outcomes | Carlin, 2021 | Moderate |
| Chen X, 2025, China | SR and MA  Experimental (RCTs, quasi-experimental) | Children and adolescents (10-18) - normal weight, overweight/obese | Jan 2012-Mar 2024 | 6 | Wearables (accelerometers, pedometers) | PA | 15 | 2 - PA outcomes, RCTs | Baldursdottir, 2017 Ridgers, 2021 | Moderate |
| Creaser AV, 2021, UK | SR  Experimental (any), observational (any) | Children and adolescents (5-19) | <Dec 2019 | 6 | Wearables (wearables) | PA | 33 | 2 - evaluation of effectiveness, healthy, RCTs | Guthrie, 2015 Ruotsalainen, 2015 | Critically low |
| De Luca V, 2025, Italy | SR  Experimental (any), observational (any) | Adolescents (12-17) | 2013-Feb 2023 | 2 | Mix - mobile apps, wearable activity trackers, gamification, social media, web-based telecoaching system (mobiles, telephones) | Multiple health behaviors (PA, weight, diet) | 20 | 2 - PA outcomes, RCTs | Verswijveren, 2022 Ridgers, 2021 | High |
| Dobbie LJ, 2022, UK | SR  Experimental (any) | All ages | NR | NR | Mix (mix) | PA and weight/obesity/body composition | 7 | 1 - age <18 | Chen, 2019 | Critically low |
| Emberson MA, 2021, USA | SR  Experimental (RCTs) | All ages | 2013-2020 | 7 | Mix - apps, gamification, platforms, social media (smartphones) | PA | 20 | 2 - age <18 | Direito, 2015 Smith, 2014 | Critically low |
| França, C, 2022, Portugal | SR  Experimental (any), observational (any) | Adolescents (12-19) | May 2012-May 2022 | 3 | Mix (mix) | Multiple health behaviors | 13 | 1 - PA outcomes | Pope, 2018 | Moderate |
| Ha T, 2025, USA | SR  Experimental (RCTs, quasi-experimental) | Children and adolescents (6-19) | <May 2024 | 7 | Mix - active video games, web-based platforms, mobile apps, social media (accelerometers, pedometers, smartphones) | PA | 58 | 4 - PA outcomes, RCTs | Layne, 2022 Lee, 2012 Petrušič., 2022 Robertson, 2018 | Moderate |
| He Z, 2021, China | SR and MA  Experimental (any) | Children and adolescents (6-18) | <Jun 2020 | 5 | Mix - apps, text messaging, exergames (smartphones) | PA | 9 | 5 – healthy, PA outcomes | Garde, 2015 Garde, 2016 Garde, 2018 Direito, 2015 Thompson, 2016 | Moderate |
| Jacob CM, 2021, UK | SR and MA  Experimental (any) | Adolescents (10-19) - high-income countries | 2006-2020 | 4 | Mix - apps, websites, CD-ROM, text messages, social media, computer counseling, games (mobiles, wearables, computers) | Weight | 33 | 5 - digital tools for delivery, published >2009, RCTs | Pfeiffer, 2019 Dewar, 2013 Lubans, 2016 Smith, 2014 Ezendam, 2012 | Moderate |
| Kassim PSJ, 2025, Malaysia | SR and MA  Experimental (RCTs, quasi-experimental) | Adolescents (10-19) - overweight/obese | <Apr 2024 | 7 | Mix -smartphone apps, websites, wearables, exergames, text messaging, social media (accelerometers, pedometers, heart rate monitors) | PA | 18 SR (13 MA) | 3 - published >2009, RCTs | Maloney, 2012 Ruotsalainen, 2015 Smith, 2014 | Moderate |
| Kemp BJ, 2021, UK | SR  Experimental (RCTs) | Adolescents (10-17) | <Apr 2020 | 5 | Mix - apps, website, CD-ROM, text messages, social media, computer counseling, games (mobiles, wearables, computers, tablets) | Multiple health behaviors (PA, diet, alcohol consumption), weight | 7 | 1 - PA outcomes | Chen, 2011 | Moderate |
| Klos L, 2020, Germany | SR  Experimental (any), observational (any) | Children, Adolescents and Young Adults | Sep 2009 - Apr 2019 | 2 | Mix - Internet, social media, exergames (mix) | PA | 13 | 1 - age >5, digital tools for delivery, RCTs | Direito, 2015 | Critically low |
| Lam C, 2022, UK | SR  Experimental (any), observational (any) | Children and adolescents | >2010 | 6 | Mix (mix) | Weight | 23 | 2 – healthy, RCTs | Direito, 2015 Ridgers, 2017^a^ | Critically low |
| Lamas S, 2023, Portugal | SR  Experimental (any) | Children and adolescents | 2003-2021 | 5 | Gamification tools - serious games (mix) | PA and diet | 26 | 2 - RCTs | Baranowski, 2011 Baranowski, 2019 | Critically low |
| Langarizadeh M, 2021, Iran | SR  Experimental (RCTs, quasi-experimental) | Children and adolescents (<19) | <Dec 2020 | 5 | Apps (mobiles) | Weight | 9 | 1 - age >6, PA outcomes, RCTs | Smith, 2014 | Critically low |
| Lee YS, 2022, Singapore | SR  Experimental (any) | All ages | 1978 - Aug 2021 | 3 | Mix (mix) | PA | 52 | 1 - age <18, digital tools for delivery, RCTs | Ngo, 2014 | Critically low |
| Li SJ, 2025, China | SR and MA  Experimental (RCTs) | All ages (7-79) | <Mar 2024 | 6 | Gamification tools -exergames (console - Sony PlayStation, Nintendo Wii, and Microsoft Xbox) | PA | 20 | 6 - age <18, healthy, published >2009 | Baranowski, 2012 Comeras-Chueca, 2022 Garde, 2016 Lau, 2016 Trost, 2014 Maloney, 2012 | Moderate |
| Longobucco Y, 2023, Italy | SR  Experimental (any) | Children and adolescents (6-18) | <Mar 2022 | 4 | Mix (mix) | PA and SB | 7 | 1 - digital tools for delivery | Robbins, 2019 | Critically low |
| Love R, 2019, UK | SR and MA  Experimental (RCTs) | Children and adolescents (6-18) | <Feb 2017 | 6 | Mix (mix) | PA | 25 SR (17 MA) | 2 - digital tools for delivery | Smith, 2014 Dewar, 2013 | Moderate |
| Ludwig K, 2018, UK | SR  Experimental (RCTs, quasi-experimental) | Children and adolescents (10-19) | <Nov 2017 | 4 | Text messaging (mobiles) | PA and SB | 13 | 4 - RCTs | Brannon, 2017 Dewar, 2013 Dewar, 2014 Lubans, 2012 | Critically low |
| Mazeas A, 2022, France | SR and MA  Experimental (RCTs) | All ages (9-73) | 2010-2020 | 5 | Gamification tools - game elements in devices (mix) | PA | 18 SR (16 MA) | 5 - age <18, healthy, no feasibility studies | Direito, 2015 Garde, 2015 Garde, 2016 Garde, 2018 Leinonen, 2017 | Moderate |
| Na A, 2020, Malaysia | SR  Experimental (RCTs, pre-post), observational (any) | Children and adolescents (7-18) | Jan 2013-Dec 2017 | 5 | Gamification tools -exergames (consoles) | Obesity | 6 | 2 - RCTs | Staiano, 2017 Trost, 2014 | Moderate |
| Nash EA, 2021, Qatar/UK | SR  Experimental (RCTs, quasi-experimental) | Children and adolescents (>5), adults | Jan 1985 - Nov 2020 | 6 | Mix - apps, wearables, text messages, computer prompts (mix) | PA | 14 | 1 - age <19, RCTs | Allafi, 2020 | Critically low |
| Oliveira CB, 2020, Brazil | SR and MA  Experimental (RCTs, quasi-experimental) | Children and adolescents (2-19) | <Oct 2018 | 5 | Gamification tools -exergames (mix) | PA and weight/obesity/body composition | 12 | 5 - published >2009 | Baranowski, 2012 Duncan, 2010 Graves, 2010 Maddison, 2011 Staiano, 2017 | Moderate |
| Rodrigo-Sanjoaquín J, 2022, Spain | SR and MA  Experimental (any) | Children (5-12) | Jan 2010 - Dec 2021 | 5 | Wearables (wearables) | PA and SB | 24 | 1 - digital tools for delivery, PA outcomes, RCTs | Morris, 2019 | Moderate |
| Schwarz A, 2023, Netherlands | SR  Experimental (any) | Children and adolescents (4-18) | <Jun 2022 | 4 | Apps (mobiles) | PA | 21 | 3 - PA outcomes, RCTs | Direito, 2015 Leinonen, 2017 Seah, 2021 | Critically low |
| Seims AL, 2023, UK | SR  Experimental (any) | Children and adolescents (2-16) | <Jun 2022 | 9 | Gamification tools -exergames, + coaching/telehealth (mix) | PA | 13 | 6 - healthy, published >2009, RCTs | Baranowski, 2012 Graves, 2010 Maddison, 2011 Rhodes, 2017 Errickson, 2012 Staiano, 2018 | Critically low |
| Sequí-Domínguez I, 2024, Spain | SR and MA  Experimental (RCTs) | Children and adolescents (5-17) | <Feb 2022 | 4 | Mix - apps, exergames, telephone-based, text messages, web-based (mix) | PA | 20 | 13 - healthy, PA outcomes, published >2009 | Direito, 2015 Staiano, 2017 Staiano, 2018 Babic, 2016 Dewar, 2013, Dewar, 2014 Lubans, 2012 Lubans, 2016 Smith, 2014 Ruotsalainen, 2015 Thompson, 2016 Ezendam, 2012 Guagliano, 2020 | Moderate |
| Shin Y, 2019, Korea | SR and MA  Experimental (RCTs, pre-post) | Adolescents (10-19) | 2013-2018 | 6 | Mix (mobiles) | Multiple health behaviors | 11 | 5 - PA outcomes, RCTs | Babic, 2016 Direito, 2015 Lubans, 2016 Smith, 2014 Thompson, 2016 | Moderate |
| Solar Figueroa VA, 2025, Chile | SR  Experimental (RCTs) | Children and adolescents (6-18) | 2013-Aug 2023 | 4 | Gamification tools – exergames (console) | PA and weight/obesity/body composition | 10 | 2 - PA outcomes | Comeras- Chueca, 2022 Staiano, 2018 | Moderate |
| Spring FDH, 2025, Denmark | SR  Experimental (any) | Children and adolescents (0–18) - overweight/obese | NR | 2 | Gamification tools – exergames (console) | PA and weight/obesity/body composition | 12 | 3 - PA outcomes | Comeras- Chueca, 2022 Trost, 2014 Staiano, 2018 | Moderate |
| Stecher C, 2023, Germany/USA | SR and MA  Experimental (RCTs) | All ages | <Apr 2020 | 4 | Apps (mobiles) | PA | 22 | 4 - age <18 | Direito, 2015 Garde, 2018 Leinonen, 2017 Robertson, 2018 | Moderate |
| Wang JW, 2024, China | SR and MA  Experimental (RCTs) | Preschoolers, children and adolescents (3-18) | <Dec 2023 | 4 | Apps (smartphones, tablets) | PA and physical fitness | 28 SR (26 MA) | 8 - age 6-17, PA outcomes | Garde, 2016 Lubans, 2016 Smith, 2014 Garde, 2018 Direito, 2015 Ridgers, 2021 Seah, 2020 Tugault-Lafleur, 2021 | Moderate |
| Wang M, 2025, China | SR and MA  Experimental (RCTs) | Preschoolers, children, adolescents (1-18) | 2010-Aug 2024 | 5 | Gamification tools (wearables, mobile apps, game platforms) | PA and SB | 16 | 9 - age 6-17, PA outcomes | Maloney, 2012 Garde, 2015 Garde, 2016 Garde, 2018 Staiano, 2016 Leinonen, 2017 Direito, 2015 Tugault-Lafleure, 2023 Seah, 2020 | Moderate |
| Wang Q, 2025, China | SR and MA  Experimental (RCTs) | Children, adolescents, adults (8-71) | 2010-Oct 2024 | 4 | Chatbot-based exercise (voice assistant devices, computers, smartphones) | PA and SB | 12 | 2 - age 6-17, PA outcomes | Tugault-Lafleur, 2023 Carlin, 2021 | Moderate |
| Wang W, 2025, China | SR and MA  Experimental (RCTs) | Children and adolescents (3-18) | <Jan 2025 | 5 | Wearables (step-count monitors) | PA and SB | 34 | 14 - healthy, published >2009 | Lubans, 2011 Manley, 2014 Smith, 2014 Morgan, 2014 Morgan, 2019 Guagliano, 2020 Duncan, 2024 Wunsch, 2024 Caillaud, 2022 Morgan, 2022 Allafi, 2020 Thompson, 2016 Verswijveren, 2022 Baldursdottir, 2015 | Moderate |
| Williams WM, 2020, USA | SR  Experimental (RCTs) | Adolescents (12-19) | 2007-2019 | 6 | Gamification tools – exergames (mix) | PA | 6 | 2 - PA outcomes, published >2009 | Pope, 2018 Maloney, 2012 | Critically low |
| Xu L, 2022, China | SR  Experimental (any) | All ages | <Dec 2020 | 6 | Gamification tools - game elements in devices (mobiles) | PA | 50 | 3 - age <18, evaluation of effectiveness, PA outcomes, RCTs | Direito, 2015 Guthrie, 2015 Pope, 2018 | Critically low |
| Yang Y, 2022, Germany | SR and MA  Experimental (RCTs, pre-post) | All ages | Jan 2018 - Aug 2021 | 5 | Gamified apps (smartphones) | PA | 19 SR (16 MA) | 2 - age <18 | Direito, 2015 Leinonen, 2017 | High |
| Yau KW, 2022, Canada | SR  Experimental (any) | Children (8-12) | Jan 2008 - Jul 2021 | 5 | Apps (mobiles) | Multiple health behaviors (PA, SB, diet) and obesity | 13 | 2 - PA outcomes, RCTs | Smith, 2014 van Woudenberg, 2018 | Critically low |

^a^ This study has a protocol design, so we considered for the analysis the paper reporting the effectiveness, published in 2021 from Ridgers et al.

Acronyms. MA: meta-analysis; PA: physical activity; NR: not reported; RCT: randomized controlled trial; SB: sedentary behavior; SD: standard deviation; SR: systematic review; UK: United Kingdom
