# Supplementary material for: Digital Tools’ Effectiveness on Physical Activity Outcomes in Children and Adolescents: Umbrella Review
Source: JMIR Public Health Surveill. 2026 Mar 24;12:e75769. doi: 10.2196/75769 (PMC13013097; doi:10.2196/75769)

**Figure S1.** Summary of the characteristics of the retrieved systematic reviews or meta-analyses (in percentage frequencies). MA: meta-analysis; PA: physical activity; RCT: randomized controlled trial; SB: sedentary behavior; SR: systematic review.


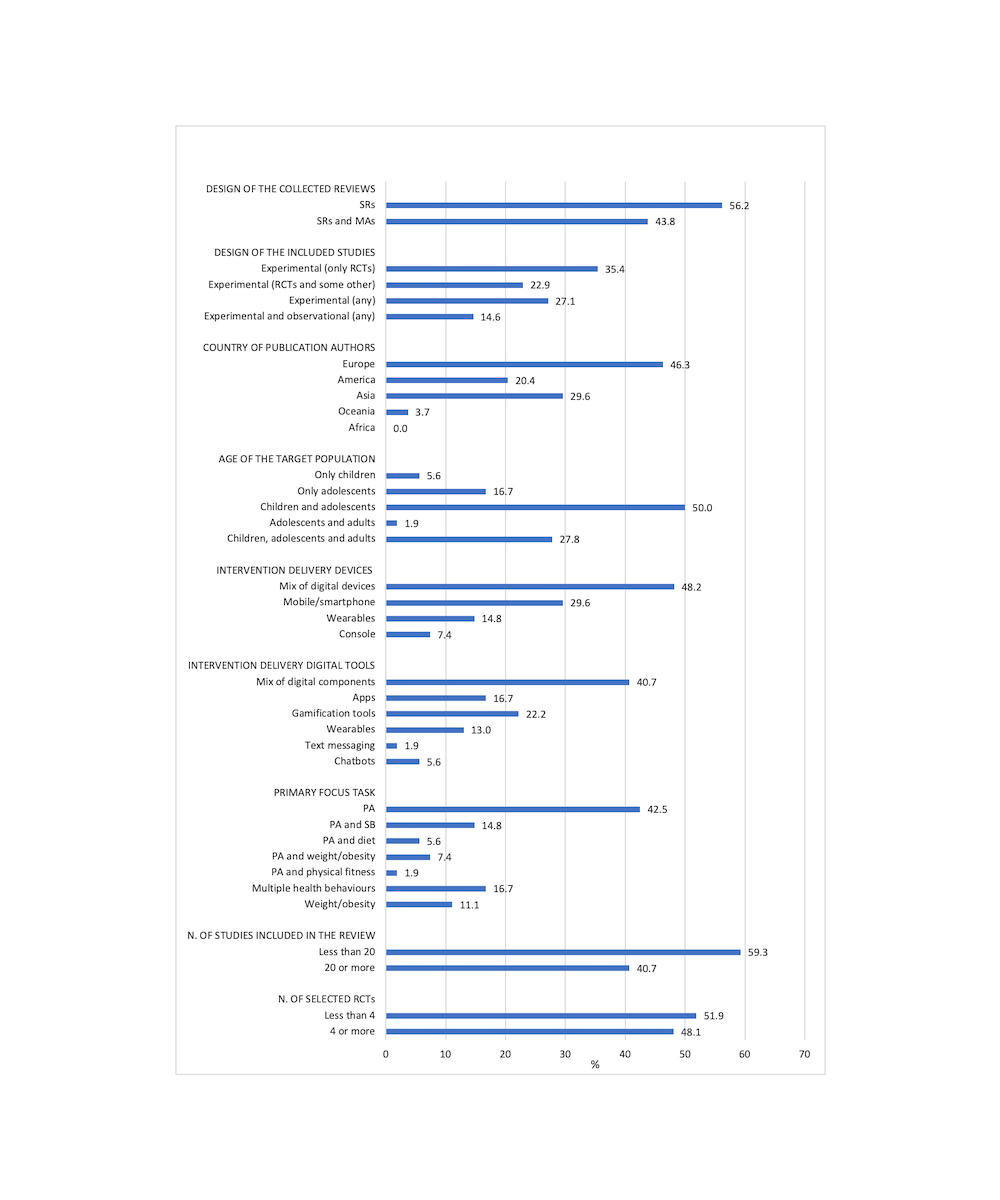


**Figure S2.** Percentage frequencies of the RCTs’ general characteristics. PA: physical activity; RCT: randomized controlled trial.


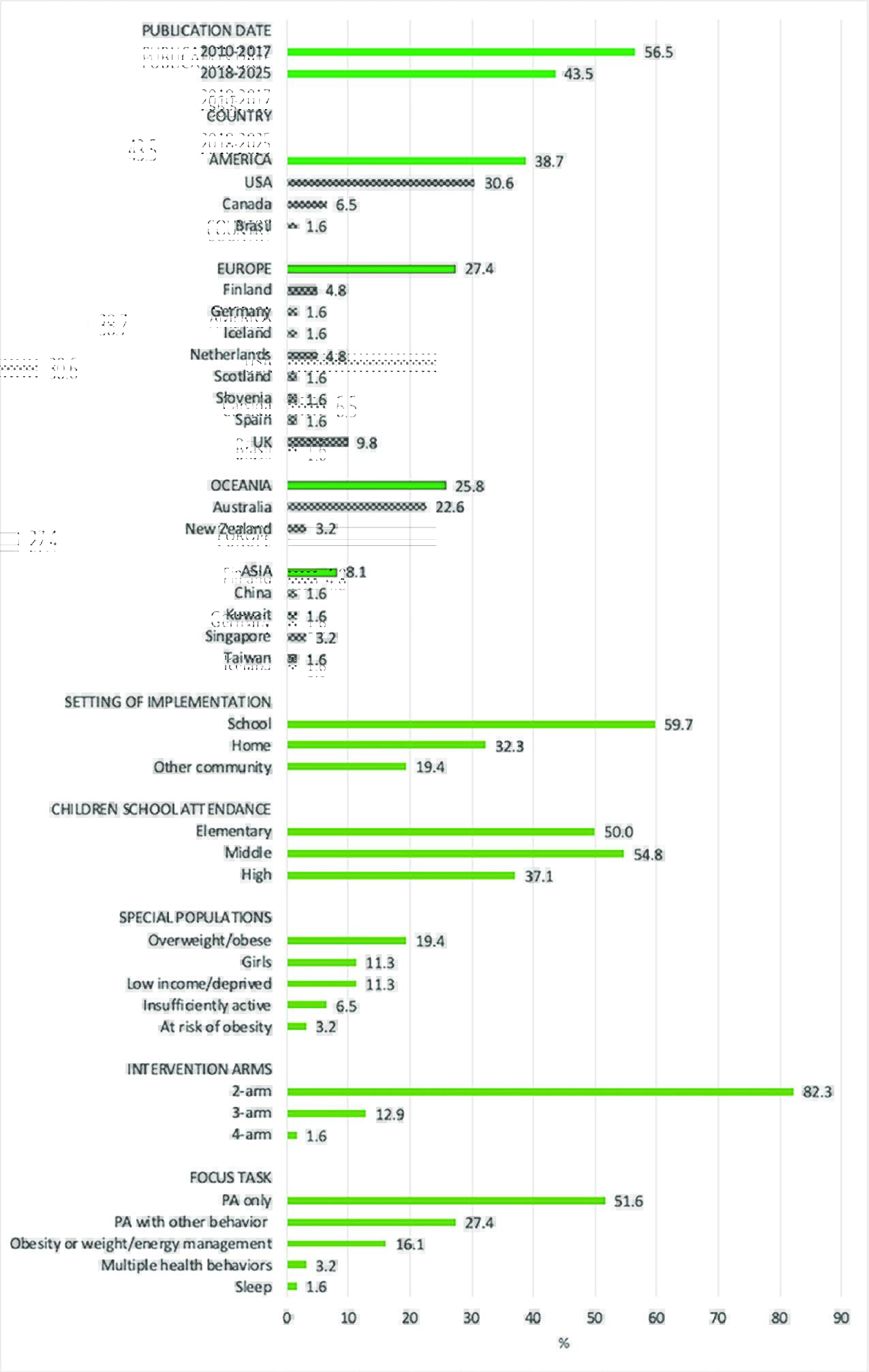


**Figure S3.** Percentage frequencies of the aspects related to the digital characteristics and effectiveness of the considered RCTs. MVPA: moderate-to-vigorous physical activity; PA: physical activity; RCT: randomized controlled trial; SB: sedentary behavior.


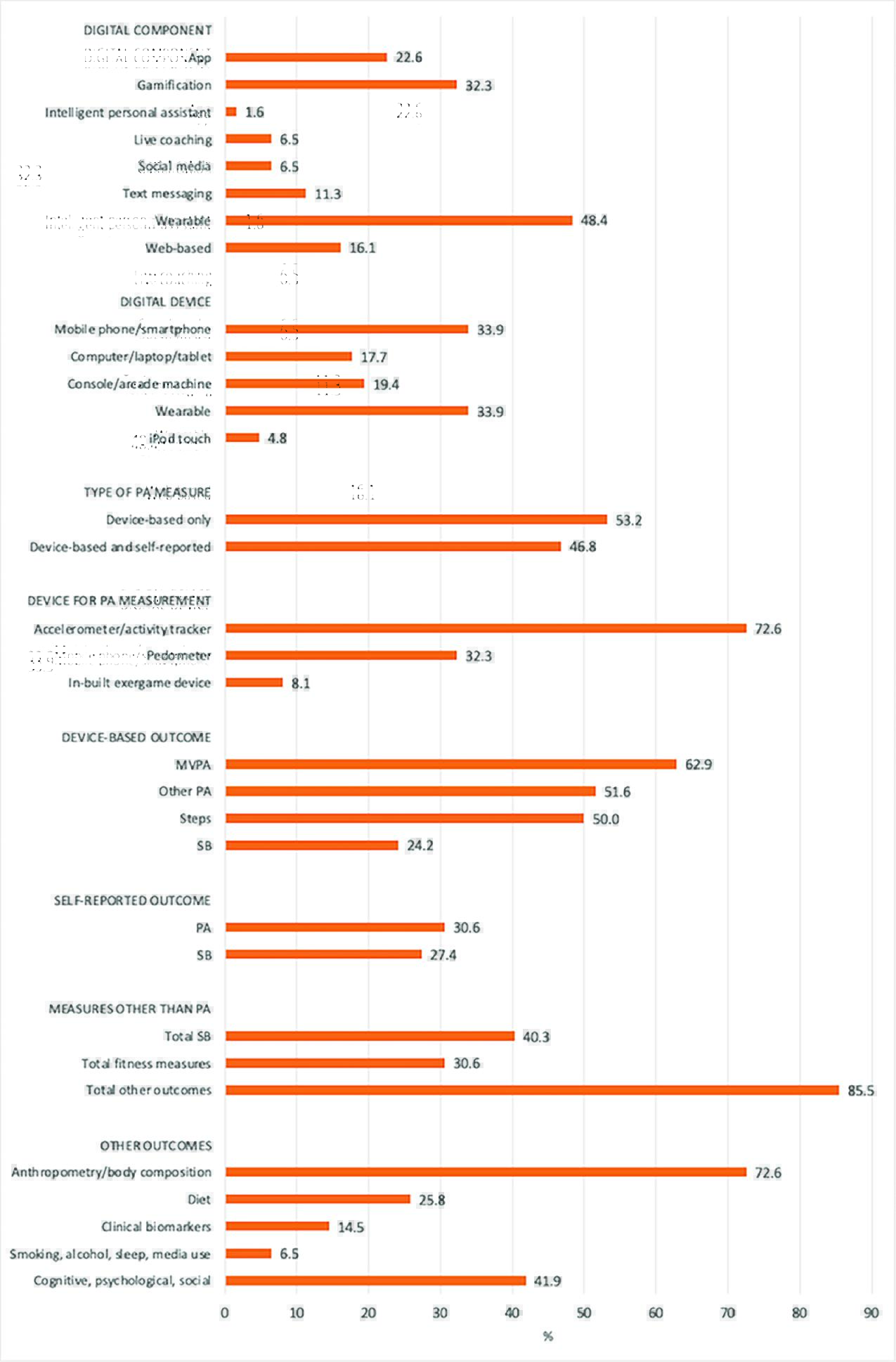

Supplement: Multimedia Appendix 3 — Figures in percentage frequencies. [file publichealth-v12-e75769-s003.docx]
