# Supplementary material for: Digital Tools’ Effectiveness on Physical Activity Outcomes in Children and Adolescents: Umbrella Review
Source: JMIR Public Health Surveill. 2026 Mar 24;12:e75769. doi: 10.2196/75769 (PMC13013097; doi:10.2196/75769)
Supplement: Multimedia Appendix 4 — AMSTAR 2 quality rating table of the selected systematic reviews and meta-analyses. AMSTAR 2: A Measurement Tool to Assess Systematic Reviews. [file publichealth-v12-e75769-s004.docx]

AMSTAR 2 Quality rating table

| **First Author, publication year** | **1. PICO** | **2. Review Methods*** | **3. Study Selection** | **4. Search Strategy*** | **5. Study Selection duplicate** | **6. Data Extraction** | **7. Excluded Studies*** | **8. Describe Studies** | **9. ROB Tool*** | **10. Report Funding** | **11. Statistical Methods*** | **12. ROB Assessment** | **13. ROB Discussion*** | **14. Study Differences** | **15. Publication Bias*** | **16. COI and Funding** | **AMSTAR 2 Quality Rating** |
| --- | --- | --- | --- | --- | --- | --- | --- | --- | --- | --- | --- | --- | --- | --- | --- | --- | --- |
| Au WW, 2024 | Yes | Yes | Yes | Yes | No | Yes | No | Yes | Yes | Yes | Yes | Yes | Yes | Yes | Yes | Yes | Moderate |
| Baumann H, 2022 | Yes | Yes | Yes | Partial Yes | No | Yes | No | Partial Yes | Yes | No | Yes | Yes | Yes | Yes | Yes | Yes | Moderate |
| Böhm B, 2019 | Yes | Partial Yes | Yes | Partial Yes | Yes | Yes | No | Partial Yes | No | No | n/a | n/a | No | No | n/a | Yes | Critically low |
| Bonvicini L, 2022 | Yes | Yes | No | Partial Yes | Yes | Yes | No | Yes | Yes | No | n/a | n/a | Yes | Yes | n/a | Yes | Critically low |
| Casado-Robles C, 2022 | No | Yes | No | Partial Yes | Yes | Yes | No | Yes | No | No | Yes | Yes | No | Yes | Yes | Yes | Critically low |
| Chai LK, 2022 | No | Partial Yes | Yes | Partial Yes | Yes | Yes | No | Partial Yes | Partial Yes | Yes | n/a | n/a | Yes | Yes | n/a | Yes | Critically low |
| Champion KE, 2019 | No | Yes | No | Partial Yes | Yes | Yes | No | Yes | No | No | Yes | Yes | Yes | Yes | No | Yes | Critically low |
| Chen J, 2025 | Yes | Yes | Yes | Yes | Yes | Yes | No | Yes | Yes | Yes | n/a | n/a | Yes | Yes | n | Yes | Moderate |
| Chen X, 2025 | Yes | Yes | Yes | Yes | Yes | Yes | No | Yes | Yes | Yes | Yes | Yes | Yes | Yes | Yes | Yes | Moderate |
| Creaser AV, 2021 | Yes | Partial Yes | Yes | Partial Yes | Yes | Yes | No | Partial Yes | Yes | Yes | n/a | n/a | Yes | Yes | n/a | Yes | Critically low |
| De Luca V, 2025 | Yes | Yes | No | Partial Yes | Yes | Yes | No | Yes | Yes | Yes | n/a | n/a | Yes | Yes | n | Yes | High |
| Dobbie LJ, 2022 | No | No | No | No | No | No | No | Yes | No | No | n/a | n/a | Yes | Yes | n/a | Yes | Critically low |
| Emberson MA, 2021 | Yes | Partial Yes | Yes | Partial Yes | Yes | Yes | No | Yes | Yes | No | n/a | n/a | No | No | n/a | Yes | Critically low |
| França, C, 2022 | Yes | Yes | Yes | Yes | Yes | Yes | No | Partial Yes | Yes | Yes | n/a | n/a | Yes | Yes | n/a | Yes | Moderate |
| Ha T, 2025 | Yes | Yes | Yes | Yes | Yes | Yes | No | Yes | Yes | Yes | n/a | n/a | Yes | Yes | n/a | Yes | Moderate |
| He Z, 2021 | No | Yes | Yes | Partial Yes | Yes | Yes | No | Partial Yes | Yes | No | Yes | Yes | Yes | Yes | Yes | Yes | Moderate |
| Jacob CM, 2021 | No | Yes | Yes | Partial Yes | Yes | Yes | No | Partial Yes | Partial Yes | No | Yes | Yes | Yes | Yes | Yes | Yes | Moderate |
| Kassim, 2025 | Yes | Yes | Yes | Yes | Yes | Yes | No | Yes | Yes | Yes | Yes | Yes | Yes | Yes | Yes | Yes | Moderate |
| Kemp BJ, 2021 | Yes | Yes | No | Yes | Yes | Yes | No | Yes | Yes | Yes | n/a | n/a | Yes | Yes | n/a | Yes | Moderate |
| Klos L, 2020 | No | No | Yes | Partial Yes | Yes | Yes | No | Partial Yes | No | No | n/a | n/a | No | Yes | n/a | Yes | Critically low |
| Lam C, 2022 | Yes | No | Yes | Partial Yes | Yes | No | No | Partial Yes | Partial Yes | No | n/a | n/a | Yes | Yes | n/a | Yes | Critically low |
| Lamas S, 2023 | No | No | Yes | Partial Yes | Yes | Yes | No | Yes | No | No | n/a | n/a | No | No | n/a | Yes | Critically low |
| Langarizadeh M, 2021 | Yes | No | Yes | Partial Yes | Yes | Yes | No | Yes | No | No | n/a | n/a | No | Yes | n/a | Yes | Critically low |
| Lee YS, 2022 | Yes | Yes | Yes | Partial Yes | No | No | No | Yes | Yes | No | n/a | n/a | Yes | Yes | n/a | Yes | Critically low |
| Li SJ, 2025 | Yes | Yes | Yes | Yes | Yes | Yes | No | Yes | Yes | Yes | Yes | Yes | Yes | Yes | Yes | Yes | Moderate |
| Longobucco Y, 2023 | Yes | Yes | Yes | Partial Yes | Yes | Yes | No | Yes | Yes | No | n/a | n/a | Yes | Yes | n/a | Yes | Critically low |
| Love R, 2019 | Yes | Yes | Yes | Partial Yes | Yes | Yes | No | No | Yes | No | Yes | Yes | Yes | Yes | Yes | Yes | Moderate |
| Ludwig K, 2018 | Yes | Yes | Yes | Partial Yes | Yes | Yes | No | Partial Yes | Yes | No | n/a | n/a | Yes | Yes | n/a | Yes | Critically low |
| Mazeas A, 2022 | Yes | Yes | Yes | Partial Yes | Yes | Yes | No | Partial Yes | Yes | No | Yes | Yes | Yes | Yes | Yes | Yes | Moderate |
| Na A, 2020 | Yes | Partial Yes | Yes | Partial Yes | Yes | Yes | No | Yes | Yes | Yes | n/a | n/a | Yes | Yes | n/a | Yes | Moderate |
| Nash EA, 2021 | Yes | Yes | Yes | Partial Yes | Yes | Yes | No | Yes | Yes | No | n/a | n/a | Yes | Yes | n/a | No | Critically low |
| Oliveira CB, 2020 | Yes | Yes | Yes | Partial Yes | Yes | Yes | No | No | Yes | No | Yes | Yes | Yes | Yes | Yes | Yes | Moderate |
| Rodrigo-Sanjoaquín J, 2022 | Yes | Yes | Yes | Partial Yes | Yes | Yes | No | Yes | Yes | No | Yes | Yes | Yes | Yes | Yes | Yes | Moderate |
| Schwarz A, 2023 | Yes | Yes | Yes | Partial Yes | Yes | Yes | No | No | Yes | No | n/a | n/a | Yes | Yes | n/a | Yes | Critically low |
| Seims AL, 2023 | Yes | Yes | Yes | Partial Yes | Yes | Yes | No | Yes | Yes | No | n/a | n/a | Yes | Yes | n/a | Yes | Critically low |
| Sequí-Domínguez I, 2024 | Yes | Yes | Yes | Yes | Yes | Yes | No | Yes | Yes | Yes | Yes | Yes | Yes | Yes | Yes | Yes | Moderate |
| Shin Y, 2019 | Yes | Yes | Yes | Partial Yes | Yes | Yes | No | Yes | Yes | No | Yes | Yes | Yes | Yes | Yes | Yes | Moderate |
| Solar Figueroa VA, 2025 | Yes | Yes | Yes | Yes | Yes | Yes | No | Yes | Yes | Yes | n/a | n/a | Yes | Yes | n/a | Yes | Moderate |
| Spring FDH, 2025 | Yes | Yes | Yes | Yes | Yes | Yes | No | Yes | Yes | Yes | n/a | n/a | Yes | Yes | n/a | Yes | Moderate |
| Stecher C, 2023 | Yes | Yes | Yes | Partial Yes | Yes | Yes | No | Yes | Yes | No | Yes | Yes | Yes | Yes | Yes | Yes | Moderate |
| Wang JW, 2024 | Yes | Yes | Yes | Yes | Yes | Yes | No | Yes | Yes | Yes | Yes | Yes | Yes | Yes | Yes | Yes | Moderate |
| Wang M, 2025 | Yes | Yes | Yes | Partial Yes | Yes | Yes | No | Yes | Yes | Yes | Yes | Yes | Yes | Yes | Yes | Yes | Moderate |
| Wang Q, 2025 | Yes | Yes | Yes | Partial Yes | Yes | Yes | No | Yes | Yes | Yes | Yes | Yes | Yes | Yes | Yes | Yes | Moderate |
| Wang W, 2025 | Yes | Yes | Yes | Partial Yes | Yes | Yes | No | Yes | Yes | Yes | Yes | Yes | Yes | Yes | Yes | Yes | Moderate |
| Williams WM, 2020 | Yes | Yes | Yes | Partial Yes | No | No | No | Partial Yes | Yes | No | n/a | n/a | Yes | No | n/a | Yes | Critically low |
| Xu L, 2022 | No | Yes | Yes | Partial Yes | Yes | Yes | No | Partial Yes | Yes | No | n/a | n/a | Yes | Yes | n/a | Yes | Critically low |
| Yang Y, 2022 | Yes | Yes | Yes | Partial Yes | Yes | Yes | Yes | No | Yes | No | Yes | Yes | Yes | Yes | Yes | Yes | High |
| Yau KW, 2022 | No | Yes | Yes | Partial Yes | No | No | No | Yes | Yes | No | n/a | n/a |  |  | n/a | Yes | Critically low |
