# Supplementary material for: Digital Tools’ Effectiveness on Physical Activity Outcomes in Children and Adolescents: Umbrella Review
Source: JMIR Public Health Surveill. 2026 Mar 24;12:e75769. doi: 10.2196/75769 (PMC13013097; doi:10.2196/75769)
Supplement: Multimedia Appendix 5 — Characteristics of the included randomized controlled trials. [file publichealth-v12-e75769-s005.docx]

| **First author, year, country** | **Intervention name** | **Age (yrs or mean/SD), school attendance (special population)** | **Sample dimension (M, F)** | **Intervention duration** | **Intervention arms** | **Setting for implementation** | **Focus task** | **Theoretical foundation** | **Digital component/s of intervention** | **Non digital component/s of intervention and/or BCTs** | **Digital device for intervention delivery** | **IG(s) and CG(s)** | **PA objective measurement tool** | **Objective PA/SB outcome measure (tool)** | **Subjective PA/SB outcome measure (tool)** | **Fitness tests assessed (test)** | **Other outcomes** | **Effectiveness on the different outcomes** | **Overall effectiveness** | **Risk of bias** |
| --- | --- | --- | --- | --- | --- | --- | --- | --- | --- | --- | --- | --- | --- | --- | --- | --- | --- | --- | --- | --- |
| Allafi, 2020, Kuwait | NA | 9-11, Elementary + Middle | 225 tot (110M, 115F): IG1 85; IG2 68; CG 72 | NA | 3-arm | School | PA | NA | Wearable (Yamax pedometer) | Goals, incentives | Wearable (Yamax pedometer) | IG1 (=FB group) received info on pedometers results; IG2 (=FB+R group) info on pedometers + incentive; CG use but no info on pedometers results | Pedometer (Yamax) | Steps (Yamax Digiwalker SW-200 pedometer) | None | Fitness (NA) | BMI | Effective on ObjSteps | Effective | Low |
| Babic, 2016, Australia | S4HM (Switch-off 4 Healthy Minds) | Mean 14.4, SD 0.6, High | 322 tot (111M, 211F): IG167 (60M, 107F); CG 155 (51M, 104F) | 6 months | 2-arm | School | PA, SB, mental health, BMI | Self-Determination Theory | Messaging (eHealth messaging), parental newsletters | Interactive seminar, behavioral contract | NA | IG (S4HM= eHealth messaging twice/week from social media and messaging systems (i.e., Twitter, Facebook, Kik, email or text messages); CG usual behaviors and school curriculum | Accelerometer (GENEActiv) | MVPA (GENEActiv wrist worn accelerometer) | Screen-time (=TV, video/DVD, computer, tablet/ smartphone) (Adolescent Sedentary Activity Questionnaire -ASAQ) | None | BMI, mental health (well-being, psychological distress, self-perceptions, motivation) | Non effective on MVPA | Non effective | Some concern |
| Baldursdóttir, 2017, Iceland | NA | 15-16, High | 53 tot: IG 26 (44%M, 56%F); CG 27 (34.6%M, 65.4%F) | 3 weeks | 2-arm | School | Sleep | NA | Wearable (Yamax pedometer, hip-worn) | PA advices, step diary | Wearable (Yamax pedometer) | IG pedometers and step diaries; CG no pedometers and step diaries | Pedometer (Yamax) | Steps (Yamax CW-701 pedometer) | PA frequency during leisure time (step diaries) | Fitness (NA) | Smoking, alcohol, sleep, media use | Effective on ObjSteps | Effective | Low |
| Baranowski, 2011, USA | Diab and Nano study | 10-12, Elementary + Middle (normal and overweight) | 153 tot (86M, 67F): IG 103 (58M, 45F); CG 50 (28M, 22F) | 2 months | 2-arm | Home | PA, diet | NA | Gamification (role-playing videogames "Escape from Diab" and "Nanoswarm: Invasion from Inner Space") | Incentives | Computer/laptop | IG played Diab and Nano in sequence; CG played diet and PA knowledge-based games on popular websites | Accelerometer (Actigraph) | MVPA, LPA, Sed, CPM (Actigraph AM-7164 accelerometer) | None | None | BMI, Triceps, WC, diet (FV, nonfat vegetables, total energy) | Non effective on ObjMVPA/LPA/CPM and ObjSB. | Non effective | Low |
| Baranowski, 2012, USA | NA | 9-12, Elementary + Middle (normal and overweight) | 78 tot (51%M, 49%F) | 13 weeks | 2-arm | Home | PA | Self-Determination Theory | Gamification (exergame: Active Video Games for the Wii consolle "Active Life-Extreme Challenge", "EA Sports Active", "Dance Dance Revolution - DDR", "Wii Fit Plus", "Wii Sports") | Not used | Console | IG active video games; CG inactive video game | Accelerometer (Actigraph) | MVPA, LPA, Sed, CPM (Actigraph GT3X accelerometer - elastic belt) | None | None | BMI, Neighborhood safety | Non effective on ObjMVPA/LPA/CPM and ObjSB. | Non effective | Some concern |
| Baranowski, 2019, USA | Diab and Nano study | 10–12, Elementary + Middle (overweight/obese) | 145 tot (60%M, 40%F) | 3 months | 2-arm | Home | Health | NA | Gamification (role-playing videogames "Escape from Diab" and "Nanoswarm: Invasion from Inner Space") | Not used | Computer/laptop | IG played Diab and Nano; CG from waitlist | Accelerometer (Actigraph). Gameplay data collected over the Internet. | MVPA, VPA, MPA, LPA, Sed, tot PA (Actigraph GT3X accelerometer); gameplay data collected over the Internet | Self-reported PA data collection on preprogrammed tablets | None | BMI, fasting insulin, diet (FV intake, sweetened beverages) | Non effective on ObjMVPA/VPA/MPA/LPA/totPA, ObjSB and SubjPA. | Non effective | Some concern |
| Brannon, 2017, USA | NA | 13-18, High | 10 tot (3M, 7F) | 24 days | 2-arm | Home | PA | Cybernetic Control Theory | Text messaging | Incentives | Mobile phone | IG received PA intervention with text messaging; CG received PA intervention without text messaging | Accelerometer (Actigraph) | MVPA, Sed (Actigraph wActi Sleep-BT accelerometer). HR (Zephyr BioHarness 3.0 monitoring device + ZephyrLife mobile app) | PA (Calorie Counter and Diet Tracker within the MyFitnessPalTM mobile app) | None | BMI, WC | Effective on ObjMPVA. Non effective on ObjSB. Non effective on SubjPA. | Partially effective | Some concern |
| Caillaud, 2022, Australia | iEngage project | 10-12, Middle | 64 tot (45.8%M, 54.2%F): IG 57; CG 26 | 5 weeks | 2-arm | School | PA | NA | iEngage app, wearable (activity tracker) | Guidance to set goals, self-monitor, assess achievements | Mobile phone | IG1 iEngage (PAQ and Misfit sensor); IG2 GENEActiv; CG2 PAQ-C | Accelerometer (GENEActiv), activity tracker (Misfit Ray©) | MVPA (GENEActiv accelerometer), steps (Misfit Ray© activity tracker) | PA (C-PAQ) | Fitness tests: 20m multistage shuttle test, flexibility, hand grip, vertical jump, agility | BMI, body fa | Effective on ObjMVPA, ObjSteps, SubjPA and SubjSteps | Effective | Some concern |
| Carlin, 2021, UK | IPAP (Intelligent Personal Assistant Project) | 5-12, Elementary + Middle | Phase 1: 16 (7M, 9F) Phase 2: 18 (10M, 8F) | 12 weeks | 2-arm | Home | PA, health | NA | Intelligent Personal Assistants (Echo dot) | Not used | Computer, mobile phone | Phase 1: IG IPAP (intelligent personal assistant + weight management project); CG no intervention. Phase 2: IG IPAP (intelligent personal assistant); CG no intervention. | Accelerometer (ActiGraph GT3) | TPA (=LPA+MPA+VPA) (ActiGraph GT3 accelerometer, waist-worn) | SB, activity habit (Family Eating and Activity Habits Questionnaire - FEAHQ-R) | None | BMI, diet | Non effective on ObjTPA | Non effective | Low |
| Chen, 2011, USA | Web ABC study (Web-Based Active Balance Childhood) | 12-15, High (normal weight and overweight, Chinese/American) | 50 tot: IG 26; CG 24 | 8 weeks | 2-arm | Any palce (home, school, library) | PA, diet, weight | Transtheoretical Model– Stages of Change, Social Cognitive Theory | Web-based (information on diet and PA + use of software "The Wok" for preparing chinese foods) + wearable (pedometer for self-monitoring) + online activity diary | Engagement on PAs , goal setting, incentives | Laptop | IG web-based group + PA sessions + pedometer; CG web-based only information | Accelerometer (Actigraph MTI/CSA 7164) | CPM (MTI/CSA 7164 Actigraph accelerometer, hip-worn) | None | None | BMI, waist and hip circumference (WHR), blood pressure, dietary intake, knowledge and self-efficacy on PA and nutrition | Effective on counts per minute of PA | Effective | Some concern |
| Comeras-Chueca, 2022, Spain | AVG intervention | 9-12, Elementary (overweight and obese) | 29 tot (16M, 13F): IG 21; CG 8 | 5 months | 2-arm | Community (school and university) | Obesity | NA | Active video game (Xbox 360® with the Kinect, the Nintendo Wii®, dance mats, and the BKOOL® interactive cycling simulator) | Multicomponent exercise on muscular fitness, PA and motor skills (3 sessions/week) | Console | IG active video games; CG usual daily activities | Accelerometer (GENEActiv) | MVPA, VPA, LPA, TPA, Sed min/day (GENEActiv accelerometer, left wrist-worn) | None | Fitness tests: Counter Movement Jump height, maximal isometric strength of knee extension and handgrip strength. | BMI, lean mass, Motor skills(TGMD3 test). | Effective on ObjLPA, ObjSed | Partally effective | Some concern |
| Dewar, 2013, Australia | NEAT Girls (Nutrition and Enjoyable Activity for Teen Girls) | Mean 13.2, SD 0.5, Middle + High (low-income) | 357 tot (357F): Baseline IG 178, CG 179; 12 month IG 141, CG 153; 24 month IG 113, CG 121 | 24 months | 2-arm | School | Obesity | Social Cognitive Theory | Text messaging,  wearable (pedometer for self-monitoring) | School program (sport, interactive seminars, nutrition workshops, lunch-time PA sessions, PA and nutrition handbooks, parent newsletters) | NA | IG following NEAT girls; CG non following NEAT girls | Accelerometer (Actigraph) | MVPA, CPM (Actigraph MTI, 7164, GT1M, GT3X accelerometer) | Screen time min/day (Adolescent Sedentary Activity Questionnaire) | None | BMI, body fat, dietary intake | Non effective on ObjMVPA/CPM. Effective on SubjSB. | Partially effective | Some concern |
| Dewar, 2014, Australia | NEAT Girls (Nutrition and Enjoyable Activity for Teen Girls) | Mean 13.2, SD 0.5, Middle + High | 358 tot (357F): Baseline IG 178, CG 179; 12 month IG 141, CG 153; 24 month IG 113, CG 121 | 12 months | 2-arm | School | Obesity | Social Cognitive Theory | Text messaging,  wearable (pedometer for self-monitoring) | School program (sport, interactive seminars, nutrition workshops, lunch-time PA sessions, PA and nutrition handbooks, parent newsletters) | NA | IG following NEAT girls; CG non following NEAT girls | Accelerometer (Actigraph) | MVPA, MPA, VPA, Sed, CPM (ActiGraph MTI, 7164, GT1M and GT3X accelerometer) | SB (Adolescent Sedentary Activity Questionnaire) | None | Self-efficacy, environmental perceptions, social support, behavioral strategies, outcome expectations and expectancies, intentions to be physically active (Questionnaire: 6 social cognitive scales for PA mediators were designed based on constructs of SCT by Bandura) | Non effective on ObjMVPA/MPA/VPA/CPM. Non effective on ObjSB. Effective on SubjSB. | Partially effective | Low |
| Direito, 2015, New Zealand | AIMFIT (Apps for IMproving FITness) intervention | 14-17, High (insufficiently active) | 51 tot (22M, 29F): IG1 17 (8M, 9F); IG2 16 (6M, 10F); CG 18 (8M, 10F) | 8 weeks | 3-arm | Home | PA, cardiorespiratory fitness | Self-Regulatory Behavior Change Techniques | Smartphone apps (Immersive app: Zombies Run! 5K training; Nonimmersive app: Get Running-Couch to 5K) | Education, social networking/forums/messaging | Smartphone, iPod touch | IG1 use of an immersive smartphone app; IG2 use of a non-immersive app; CG usual behavior | Accelerometer (Actigraph) | MVPA (ActiGraph GT1M accelerometer) | PA (PAQ-A) | Fitness - cardiorespiratory (1-mile walk/run) | Anthropometrics, perceived enjoyment of PA, perceived competence, autonomy and relatedness while exercising | Non effective on ObjMVPA and SubjPA. | Non effective | Low |
| Duncan, 2010, UK | NA | 10-11, Elementary + Middle | 30 tot (12M, 18F): IG 15 (6M, 9F); CG 15 (6M, 9F) | 6 weeks | 2-arm | School | PA | NA | Gamification (exergames: Active Video Games Nintendo Wii; Wii Sports Tennis, Sonic and Mario at the Olympics - 100 m, 110 m hurdles, and Celebrity Sports Showdown - Horse Racing) | Not used | Console | IG played exergame; CG normal activity | Accelerometer (New Lifestyles) | MVPA, steps (New Lifestyles NL 2000 accelerometer) | None | None | BMI, body fatness, HR (Polar RS400 heart rate monitor) | Effective on steps (in the short term). Non effective on ObjMVPA. | Partially effective | High |
| Duncan, 2024, UK | NA | 6-7, Elementary | 64 tot (37M, 27F): IG 32; CG 32 | 10 weeks | 2-arm | School | PA, health | NA | Wearable (New Lifestyles NL-1000 accelerometer) | Not used | Wearable (New Lifestyles NL-1000 accelerometer) | IG accelerometer intervention; CG regular school-based activity | Accelerometer (New Lifestyles NL-1000), Accelerometer (ActiGraph GT9X) | LPA, MPA, VPA, Sed (ActiGraph GT9X accelerometer, non-dominant wrist-worn); steps (New Lifestyles NL-1000 accelerometer) | None | None | BMI, body fat, blood pressure, motor skills from TGMD3, well-being, perceived physical competence | Non effective on ObjPAs | Non effective | Low |
| Errickson, 2012, USA | DDR (Dance Dance Revolution) coaching protocol | 7-8, Elementary | NA | NA | NA | Home | PA | NA | Gamification (exergame Dance Dance Revolution) + coaching | Not used | Arcade machine | IG Enhanced (DDR + coaching); CG Basic (DDR without coaching) | Accelerometer (Actigraph). DDR logs. Sony Playstation®2 memory cards. | MVPA, VPA, TPA (Actigraph accelerometry, DDR logs, and Sony Playstation®2 memory cards) |  |  | None | Non effective on ObjMVPA/VPA/totPA. Effective on increasing DDR time gaming. | Partially effective | High |
| Ezendam, 2012, Netherlands | FATaintPHAT (VETisnietVET in Dutch) web-based computer-tailored intervention | 12-13, Middle | 884 tot: IG 485 (284M, 198F); CG 398 (198M, 200F) | 11 weeks | 2-arm | School | Energy balance | Theory of Planned Behavior, Precaution Adoption Process Model, implementation intentions | Web-based (website with web modules) | Not used | Computer | IG have followed 8 modules behaviors related to energy balanc; CG no intervation; | Pedometer (Yamax) | Steps (pedometers Digiwalker SW200 YAMAX) in a subsample | PA <60 min/day, >60 min/day, SB (Flemish questionnaire) | None | BMI, WC, diet (SSB, snacks, fruit, vegetables, whole-wheat bread) | Effective on Objstep. Non effective on SubjPA and SubjSB. | Partially effective | Some concern |
| Garde, 2015, USA | NA | 8-13, Elementary + Middle | 47 tot (16M, 31F): IG 26 (9M, 17F); CG 21 (7M, 14F) | 2 weeks | 2-arm | Home | PA | Self-Determination Theory | Gamification (exergame MobileKids Monster Manor - MKMM) | Not used | Smartphone | IG1 "game group", played MKMM installed in a iphone; IG2 "feedback group", received daily activity feedback (steps and active minutes) via an online program (Tractivity web-based software) | Accelerometer (Tractivity) | Steps, active minutes (Tractivity accelerometer) | None | None | BMI, attitudes (attitudes toward the activity sensor, the game, the social encouragement within gameplay, the requirement of being active to earn points, and if the game encouraged more physical activity overall) | Effective on ObjSteps and Objactiveminutes. | Effective | Some concern |
| Garde, 2016, Canada | NA | Mean 11.3, SD 1.2, Elementary + Middle | 42 tot | 4 weeks | 2-arm | School | PA | Self-Determination Theory | Gamification (exergame MobileKids Monster Manor - MKMM) | Not used | iPod touch | IG played MKMM; CG no MKMM. | Accelerometer (Tractivity) | Steps, active minutes (Tractivity accelerometer) | None | None | BMI | Effective on ObjSteps and Objactiveminutes. | Effective | Low |
| Garde, 2018, Canada | NA | Mean 10.6, SD 0.51, Elementary + Middle | 37 (21M, 16F) | 4 weeks | 2-arm | School | PA | Self-Determination Theory | Gamification (exergame MobileKids Monster Manor - MKMM) | Not used | iPod touch | IG played MKMM; CG no MKMM. | Accelerometer (Tractivity) | Steps, active minutes (Tractivity accelerometer) | None | None | BMI, other attitudes (attitudes toward the activity monitor, the game, the social encouragement within the gameplay, and if the game encouraged more physical activity overall, and one openended question for general comments) (Game Response Survey) | Effective on ObjSteps and Objactiveminutes. | Effective | Low |
| Graves, 2010, UK | NA | 8-10, Elementary | 42 tot (28M, 14F): IG 22 (13M, 9F); CG 20 (15M, 5F) | 12 weeks | 2-arm | School | PA, weight | NA | Gamification (exergames: Playstation 2, 3 and Nintendo Wii; jOG packing) + wearable (pedometer, hip-worn) | Not used | Console | IG play with two devices for home-use + hip-worn pedometer + standard console controller; CG were asked to continue playing their video games as normal | Accelerometer (Actigraph) | PA, TPA, Sed, CPM, steps (ActiGraph GT1M) | Min of sedentary (seated) video gaming, active video gaming (other than jOG step-powered gaming at 6 and 12 weeks), TV viewing, computer/internet use for pleasure, working on a computer, reading for pleasure (self-report questionnaire) and doing homework | Fitness test on treadmill (walked at 4km/h 1 fior 3 min, rested for 30 s; jogged at 8 km/h 1for 3 min. | Body fat, BMI | Non effective on ObjSteps, ObjtotPA/CPM and ObjSB. Effective on time spent in active games and SubjSB. | Partially effective | High |
| Guagliano, 2020, UK | FRESH (Families Reporting Every Step to Health) intervention | 7-11, Elementary + Middle | 82 tot (59.8%M, 40.2%F): IG1 30 (50%M, 50%F), IG2 23 (82.6%M, 17.4%F), CG 29 (51.7%M, 48.3%F). | 52 weeks | 3-arm | Home | PA | Self-Determination Theory | Web-based (FRESH Website) + wearable (Walk4Life pedometer ) | Not used | Computer, wearable (pedometer) | IG1 FAM arm (family theory intervention online to increase PA + family action planners + FRESH website to choose weekly step challenge); IG2 PED arm (pedometer + info); CG no intervention | Accelerometer (Actigraph) Pedometer (Walk4Life). | Steps (Walk4Life Pedometer). MVPA (ActiGraph GT3X accelerometer). | Screen-time (questionnaire) | Fitness - aerobic (8-min submaximal step test) | BMI, WC, QoL, family co-participation in PA, PA awareness, family social norms for PA, family support, motivation for PA, children’s perceived autonomy competence, and relatedness, economic (e.g., expenditure for PA) | Non effective on ObjSteps, ObjMVPA and SubjSB. | Non effective | Low |
| Guthrie, 2015, USA | Zamzee meter/website system | 11-14, Middle | 182 tot (94M, 88F): IG 61 (30M, 31F); CG 59 (36M, 23F) | 6 weeks | 3-arm | Home | PA | Self-Determination Theory | Web-based (website) + wearable (Zamzee activity monitor) or exergame (Dance Dance Revolution) | Not used | Computer | IG active group receiving Zamzee activity meters + Zamzee web; CG1 = a passive group receiving Zamzee activity meters; CG2 = active group receiving Zamzee activity meters and the Dance Dance Revolution (DDR) active video gam | Accelerometer (Zamzee) | MVPA (Zamzee activity meter-accelerometer) | None | None | BMI | Effective on ObjMVPA. | Effective | Some concern |
| Honas, 2024 | PAAC3 (Physical Activity Across the Curriculum 3) | 6-11, Elementary | 71 tot: IG1 54; IG2 40; IG3 38 | 24 months | 3-arm | School | PA | Social Cognitive Theory | Videos developed within PAAC3 for active break, Go Noodle© "Indoor recess" videos | Classroom teacher delivered activity break | NA | IG1 PAAC videos; IG2 PAAC in person; CG Go Noodle Indoor recess videos | Accelerometer (ActiGraph GT3X+) | PA counts/min (Actigraph GT3X+ accelerometer, waist-worn). METs (MVPA) and Kcal/min (=Intensity of activity breaks) (COSMED® K5 portable indirect calorimeter) | None | None | Height, weight | Effective on ObjMVPA and ObjCounts/min | Effective | Low |
| Jake-Schoffman, 2018, USA | mFIT (Motivating Families with Interactive Technology) study | 9-12, Elementary + Middle | 33 tot (12M, 21F): IG1 17 (9M, 8F); IG2 16 (4M, F12) | 12 weeks | 2-arm | Home | PA, diet | Social Cognitive Theory, Theory of Planned Behavior | Website (mFIT website for Food and step logs) + text messaging (on mFIT website) + app (TECH+ component) + wearable (ACCUSPLIT AX2720 pedometer for self-monitoring) | Program for activities; goals and rewards | Mobile phone | IG1 (TECh+ family-based activities with mFIT website); IG2 (TECH invididual based activity); | Accelerometer (Actigraph). Pedometer (ACCUSPLIT). | MVPA (Actigraph GT1M accelerometer). Steps (ACCUSPLIT AX2720 pedometer) | None | None | Satisfaction, family cohesion, Parent–Child Communication, Family Engagement, and Family Closeness, Dietary Self-Monitoring, Dietary Consumption | Non effective on ObjSteps and MVPA. | Non effective | Low |
| Jauho, 2015, Finland | NA | 17–18, High | 276 tot: IG 137, CG 139 | 3 months | 2-arm | Home | PA | NA | Wearable (Polar Active activity monitor, wrist-worn) | Not used | Wearable (Polar Active activity monitor) | IG wore activity monitor; CG continued their normal life | Accelerometer (Polar Active) | MVPA, LPA, Sed (Polar Active activity monitor, wrist-worn) | Sitting time h/day, PA level (questionnaire) | Fitness - grip strength (dynamometer), aerobic fitness (Polar Fitness Test) | BMI, WC, body fat, FFM, Self-perceived physical fitness, self-perceived health, smoke, alcohol use | Effective on ObjMVPA, ObjSB and SubjSB (in the short term). Non effective on ObjLPA and SubjPA. | Partially effective | Some concern |
| Lau, 2016, China | NA | 8-11, Elementary | 80 tot (55M, 25F): IG 40 (29M, 11F); CG 40 (26M, 14F) | 12 weeks | 2-arm | School | PA, aerobic fitness, psychological aspects | NA | Gamification (exergame: Xbox 360 - 10-pin bowling, boxing, track and field, table tennis, beach volleyball, and association football in Season 1 and golf, darts, baseball, skiing, tennis, and American football in Season 2; with Kinect sensor, and Depth camera) | Not used | Console | IG played Xbox 360, twice/week during after-school hours, each for 60 min over 12 weeks in duration; CG no intervention | Accelerometer (Actigraph) | MVPA, tot PA (ActiGraph GT3X+ accelerometer) | Nine items from the self-efficacy scale rate their confidence for doing PA at various intensities (i.e., light, moderate, vigorous) and durations (i.e., 10, 30, 60 minutes) per day on most of the days (at least 5 days/week) | Fitness - aerobic (20-m shuttle run) | BMI, PA task efficacy, barrier efficacy, enjoyment | Effective on ObjMVPA/totPA. | Effective | High |
| Layne, 2022, USA | NA | 9-10, Elementary | 130 tot (69M, 61F): IG1 43 (21M, 22F); IG2 41 (25M, 16F); CG 46 (23M, 23F). 45 (21M, 24F) worn accelerometer; 30 (15M, 15F) wore HRMs | NA | 3-arm | School | PA | NA | Wearable (Polar GX heart rate monitor) | Sport Education (SE) Model | Wearable (Polar GX heart rate monitor) | IG1 "SEPS group" (heart rate monitor+sport education model; IG2 "TPS group" (heart rate monitor)+traditional instructions; CG "Trad group" (traditional instructions). All groups performed the fitness test, and a subgroup wore accelerometers | Accelerometer (NA) | MVPA (accelerometer, waist-worn) | None | Fitness - 30-sec jump performance test | Height, weight | Effective on MVPA | Effective | Low |
| Lee, 2012, Taiwan | SPAA-G (School-based Physical Activity intervention for Adolescent Girls programme) | 16-20, High | 91F: IG 46; CG 45 | 12 weeks | 2-arm | School | PA | Self-efficacy theory | Wearable (pedometer, pocket, bag or clip) | Goal setting by participant, reward, social support, goal setting by a third party, and advice on goal setting | Pedometer (Omron pedometer, pocket, bag or clip) | IG wore Pedometer (pocket, bag or clip); CG no intervention | Pedometer (Omron HJ-720ITC) | Steps (Omron HJ-720ITC pedometer) | PA (International Physical Activity Questionnaire - IPAQ) | Fitness: standing long jump, sit-up, flexibility | BMI, perceived self-efficacy for exercise, perceived stress | Effective on ObjSteps | Effective | Low |
| Leinonen, 2017, Finland | MOPO study | 16-20, High | 496 tot: IG 250, CG 246 | 6 months | 2-arm | Home | PA | Transtheoretical Model of Behavior Change | Web-based (MOPOrtal: automated, gamified website mobile service) + wearable (Polar Active activity monitor, wrist-worn) | Not used | Mobile phone | IG "MOPO group" wrist-worn physical activity monitor (Polar Active) with physical activity feedback and access to a gamified Web-based mobile service; CG no intervention | Accelerometer (Polar Active) | MVPA, Sed (Polar Active accelerometer) | None | Fitness - bilateral maximal isometric grip strength (dynamometer), aerobic fitness (Polar Fitness Test using FT80 heart rate monitor (Polar Electro)) | BMI, WC, body fat, muscle mass, smoking, alcohol use | Effective on MVPA. Non effective on ObjSB. | Partially effective | Low |
| Lubans, 2011, Australia | PALs (Physical Activity Leaders program) | Mean 14.3, SD 0.6, High (low SES) | 100M | 6 months | 2-arm | School | PA | Social cognitive theory | Wearable (Yamax pedometer for self-monitoring) | School sport sessions, interactive seminars, lunch-time activities,PA and nutrition handbooks, leadership sessions | Pedometer | IG worn pedometer; CG waiting list | Pedometer (Yamax CW200) | Steps (Yamax CW200 pedometer) | None | Fitness - muscular (leg dynamometer, 90º push-up test, 7-stage sit-up test) | BMI, body fat, WC, diet | Non effective on ObjSteps | Non effective | Some concern |
| Lubans, 2012, Australia | NEAT Girls (Nutrition and Enjoyable Activity for Teen Girls) | 12-14, Middle + High (girls) | 357 tot: IG 178 only females; CG 179 | 12 months | 2-arm | School | Obesity | Social Ecological Model, incorporated policy and environmental changes | Text messaging, wearable (pedometer for self-monitoring) | School program (sport, interactive seminars, nutrition workshops, lunch-time PA sessions, PA and nutrition handbooks, parent newsletters) | NA | IG following NEAT girls; CG non following NEAT girls | Accelerometer (Actigraph) | MVPA, CPM (Actigraph MTI, 7164, GT1M, and GT3X accelerometer) | Screen time (Adolescent Sedentary Activity Questionnaire -ASAQ) | Fitness - muscular fitness (Push-up test); (Prone support test) | BMI, body fat, dietary intake (Energy), physical and global self-esteem, perceived body fatness | Non effective on ObjMVPA/CPM and SubjSB. | Non effective | Some concern |
| Lubans, 2016, Australia | ATLAS (Active Teen Leaders Avoiding Screen-time) obesity prevention program | 12-14, Middle + High (at risk of obesity) | 361 tot only male (IG 181; CG 180) | 20 weeks | 2-arm | School | Obesity | NA | Smartphone app, wearable (pedometers for self-monitoring) | Teacher professional learning; provision of fitness equipment; researcher-led seminars for students; face-toface PA sessions; lunch-time PA leadership sessions run by students; parental strategies for reducing recreational screen-time | Smartphone | IG following ATLAS intervetion; CG non following ATLAS intervation. | Accelerometer (Actigraph) | MVPA, CPM (Actigraph accelerometer GT3X) | SB (Adolescent Sedentary Activity Questionnaire -ASAQ) | Fitness - muscular fitness (90° push-up test), resistance training (Resistance Training Skills Battery (RTSB)) | BMI, WC, Sugar-sweetened beverage consumption, Motivation for school sport | Non effective on ObjMVPA/CPM and SubjSB. | Non effective | Some concern |
| Maddison, 2011, New Zealand | NA | 10-14, Middle (overweight and obese) | 323 tot: IG 116M, 207F; CG 119M, 43F | 25 weeks | 2-arm | Home | Body composition | NA | Gamification (exergame: Sony PlayStation EyeToy (a USB motion-capture camera to place a picture of the gamer on screen, which the gamer then interacts with) - upgrade version: EyeToy camera, dance mat, a selection of active video games (eg, Play3, Kinetic, Sport, and Dance Factory; Sony)) | Information about increasing PA, healthy eating, or weight loss; encourage to meet current PA recommendations. | Console | IG Sony PlaStation upgrade package; CG normal video game play and no information received. | Accelerometer (Actigraph). Active and non active video game play min/day. | MVPA, LPA, CPM (Actigraph AM7164-2.2C accelerometer, right hip). Active and non active video game play min/day. | None | Fitness - cardiorespiratory (20-m shuttle test) | BMI, WC, body fat, fat-free mass, food snacking | Non effective on ObjMVPA/LPA/CPM. Effective on time spent in active video game. | Partially effective | Low |
| Maloney, 2012, USA | DDR (Dance Dance Revolution) exergame | 9-17, Elementary + Middle + High (overweight and obese) | 64 tot (46.9%M, 53.1%F): IG 33, CG 31 | 12 weeks | 2-arm | Home | PA | NA | Gamification (exergame: Dance Dance Revolution) | Instructions, behavioural practice/rehearsal, adding objects to the environment, social support (unspecified), prompts/cues | Arcade machine | IG DDR + pedometers; CG waitlist + pedometers only | Accelerometer (Actigraph). DDR logs. Sony Playstation®2 memory cards. | MVPA, VPA, TPA (Actigraph accelerometer, DDR logs, and Sony Playstation®2 memory cards) | MVPA (self-reported) | None | BMI, body fat | Non effective on ObjMVPA/VPA/totPA. Effective on SubjPA. | Partially effective | High |
| Manley, 2014, USA | NA | 11-13, Middle (rural community) | 116 tot (57M, 59F): IG 55 (27M, 28F), CG 61 (30M, 31F) | 12 weeks | 2-arm | School | PA | NA | Wearable (Yamax pedometer) | Not used | Pedometer | IG pedometer; CG no pedometer | Pedometer (Yamax Digiwalker 200) | Steps (Yamax Digiwalker 200 pedometer) | None | Fitness - cardiorespiratory: 1-mile walk test | BMI, self-efficacy | Non effective on steps | Non effective | Some concern |
| Morgan, 2014, Australia | HDHK (Healthy Dads, Healthy Kids) | Mean 8.1, SD 2.1, Elementary | 104 tot: IG 53, CG 51 | 7 weeks | 2-arm | School | Health | Social Cognitive Theory, Family Systems Theory | Wearable (Yamax SW200 pedometer) | Booklets, self-efficacy, goals/ intention, outcome expectations, perceived facilitators and barriers to changes, and social support | Wearable (pedometer) | IG HDHK intervention; CG waitlist | Pedometer (Yamax SW200) | Steps (Yamax SW200 pedometer) | SB sitting time, screen time (min/day) (modified version of the CLASS) | None | BMI, WC, blood pressure, resting heart rate, self-reported dietary intake | Effective on ObjSteps. Non effective on SubjSB. | Partially effective | Low |
| Morgan, 2019, Australia | DADEE (Dads And Daughters Exercising and Empowered) intervention | 4-12, Elementary (girls) | 153 tot: IG 74, CG 79 | 8 weeks | 2-arm | Home | PA | NA | App (DADEE) + wearable (Yamax SW200 pedometer) | Group education and practical sessions, Daughters resources (tasks, folders), Sports skills program, Sport equipment pack | Smartphone | IG DADEE intervention (app + pedometer); CG wait list | Pedometer (Yamax) | Steps (Yamax SW200 pedometer) | Screen time (modified version of the Adolescent Sedentary Activity Questionnaire -ASAQ) | None | BMI, Fundamental movement skills (object control), HR (POLAR H7 heart rate sensor), Perceived competence, attendance, program satisfaction | Effective on ObjSteps and SubjSB. | Effective | Some concern |
| Morgan, 2022, Australia | DADEE (Dads And Daughters Exercising and Empowered) intervention | 4-12, Elementary (girls) | 189 tot: IG 95, CG 94 | 9 weeks | 2-arm | School | PA | NA | App (DADEE) + wearable (Yamax SW200 pedometer) | Group education and practical sessions, Daughters resources (tasks, folders), Sports skills program, Sport equipment pack | Smartphone | IG DADEE intervention (app + pedometer); CG wait list | Pedometer (Yamax) | Steps (Yamax SW200 pedometer) | Screen time (modified version of the Adolescent Sedentary Activity Questionnaire) | None | BMI, motor skills from the TGMD3, perceived competence, | Effective on ObjSteps | Effective | Some concern |
| Morris, 2019, UK | PAL (Pedometer-based physically active learning intervention) | 9-10, Elementary | 83 tot: IG 52 (21M 31F; CG 31 (13M,18F) | 6 weeks | 2-arm | School | PA | NA | Wearable (SW200 Digiwalker pedometer) | PA lessons (School Games Organizer) | Wearable (pedometer) | IG intervention; CG noemal school curriculum | Accelerometer (Actigraph) | MVPA, LPA, Sed (ActiGraph GT1M uniaxial accelerometers - right hip) | None | None | BMI | Effective on ObjLPA. Non effective on ObjMVPA and ObjSB. | Partially effective | Some concern |
| Ngo, 2014, Singapore | FIT (Family incentive trial) | 6-12, Elementary + Middle | 785 tot: IG 83M,64 F); CG (71M, 67F) | 9 months | 2-arm | Home | PA, myopia | NA | Wearable (Omron HJ-720ITC pedometer) | Education on PA and eye care, outdoor PA sessions, incentives. | Wearable (pedometer) | IG education on myopia and good eye care habits, structured weekend outdoor activities and incentives for children to increase their daily steps, as measured via pedometers; CG brochures and booklets, and information on the health benefits of PA. | Pedometer (Omron) | Steps (Omron HJ-720ITC pedometer) | PA outdoors h/day, PA tot h/day (WHO Myopia Risk Factor Questionnaire). PA tracked by parents (7-day diary adapted from the Child Development Supplement CDS-III 2007 time diary). | None | None | Effective on ObjSteps and SubjPA (in the short term). | Effective | Some concern |
| Petrušič, 2022, Slovenia | NA | 6-9, Elementary | 132 tot (62M, 70F): IG 72 (35M, 37F), CG 60 (27M, 33F) | NA | 2-arm | School | PA | NA | Gamification (Digital placebo video games) | Not used | Computer/laptop | IG used digital placebo games; CG used traditional physical education | Accelerometer (MOX) | MVPA, LPA, VPA (MOX MMOXX1.07 accelerometer) | None | None | None | Effective on ObjMVPA and ObjLPA. Non effective on ObjVPA. | Partially effective | Low |
| Pfeiffer, 2019, USA | GOTM (Girls on the Move) intervention | Mean 12.1, SD 0.99, Middle (girls, low active, low income) | 1519 tot only females: IG 735; CG 766 | 17 weeks | 2-arm | School | Adiposity, aerobic performance | Health Promotion Model, Self-Determination Theory | Web coaching | After-school PA club, two face-to-face motivational interviewing sessions | Computer | IG 1) 90 min. School PA club three days/week; 2) two face-to-face motivational interviewing sessions; 3) one motivational, interactive Internet-based session. | Accelerometer (Actigraph) | MVPA (ActiGraph GT3X+ accelerometer) | None | Fitness - aerobic (PACER) | BMI, body fat, cognitive and affective variables | Non effective on ObjMVPA. | Non effective | Low |
| Pope, 2018, USA | NA | 16-18, High | 75 tot (54F, 21M) | 12 weeks | 2-arm | School | PA | Social Cognitive Theory, Self-Determination Theory | Gamification (exergame: Camp Conquer) | Not used | NA | IG game players + Fitbit; CG no game players + Fitbit | Accelerometer (Fitbit Flex) | Steps, active min/day (Fitbit Flex activity monitor) | None | None | None | Non effective on ObjSteps and ObjActiveminutes. | Non effective | Some concern |
| Rhodes, 2017, Canada | NA | 10-14, Elementary + Middle (inactive) | 73 tot: IG (39F); CG (34F) | 3 months | 2-arm | Home | PA, SB | Theory of Planned Behavior | Gamification (exergame: Hoggan Health interactive video gaming system linked to a Sony Playstation3) | Not used | Console | IG exergame biking + video game system; CG standard stationary biking and watching TV | Machine of exergame (Hoggan Health) | Min of equipment usage, frequency of weekly use of the bikes (daily diary and record logs in the machine) | PA (Physical Activity Questionnaire for Older Children PAC-C) | None | BMI | Non effective on Obj minutes of equipment usage and on SubjPA. | Non effective | Some concern |
| Ridgers, 2021, Australia | RAW-PA (Raising Awareness of Physical Activity) study | 13-14, High (low income) | 275 tot (47.7, 52.3%F): IG 144 (51.4%M, 48.6%F); CG 131(43.6%M, 56.4%F) | 12 weeks | 2-arm | School | PA | Behavioural Choice Theory, Social Cognitive Theory | Fitbit Flex (+ app) + web-based resources + social media (FB) | Not used | Smartphone, wearable (activity monitor) | IG RAW-PA; CG wait list | Accelerometer (Actigraph; Fitbit Flex) | MVPA (ActiGraph GT3X+ accelerometer). Steps, PA intensities (Fitbit Flex activity monitor) | PA (questionnaire on an iPad) | None | EE, sleep duration (Fitbit Flex) | Non effective on ObjSteps, ObjMVPA and SubjPA. | Non effective | Low |
| Robbins, 2019, USA | GOTM (Girls on the Move) intervention | Mean 12.05, SD 0.99, Middle (girls) | 1,519 tot (1,519F) | 17 weeks | 2-arm | School | PA | Health Promotion Model, Self-Determination Theory | Web-based, iPad | Afterschool PA club (90-min offered 3 days/week); two motivational, individually tailored, face-to-face counseling sessions. | Tablet | IG the 3 components intervention; CG usual school offerings | Accelerometer (Actigraph) | MVPA (ActiGraph GT3X+ accelerometer) | PA (Minutes of MVPA for  7 consecutive days, including 5 weekdays and 2 weekend ) | None | BMI, Pubertal Stage | Non effective on ObjMVPA. | Non effective | Some concern |
| Robertson, 2018, Scotland | FitQuest project | 10-11, Elementary | 215 tot: IG 111 (55M, 56F), CG 104 (45M, 59F) | 5 weeks | 2-arm | School | PA | Social Cognitive Theory | Gamification (exergame: FitQuest) | Not used | Smartphone | IG played the game (on Samsung Galaxy Ace II phones) during at least 1 h of PE lessons; CG standard mandated PE lessons. | Accelerometer (New Lifestyles 1000) | MVPA, steps (New Lifestyles NL 1000 accelerometer) | None | None | Exercise self-efficacy | Non effective on ObjSteps and ObjMVPA. | Non effective | High |
| Ruotsaleinen, 2015, Finland | NA | 13-16, High (overweight and obese) | 46 tot: IG1 15 (5M, 10F); IG2 16 (5M, 11F); CG 15 (4M, 11F) | 12 weeks | 3-arm | School | PA, weight | Theory of Compliance | Social media (FB) + wearable (Polar Active accelerometer, wrist-worn) | Not used | Wearable, computer/smartphone | IG1 FB-delivered lifestyle PA counselling + self-monitoring; IG2 FB-delivered lifestyle counselling; CG no FB, no self-monitoring. | Accelerometer (Polar Active) | MVPA, VLPA, LPA, MPA, VPA, Vigorous plus PA, Sed (Polar Active activity monitor) | MVPA, screen time (Self-Reported Physical Activity and Screen Time Questionnaire). Self-reported PA (WHO Health Behaviors in School-Aged Children study). | None | BMI | Non effective on ObjMVPA/VLPA/LPA/MPA/VPA, ObjSB, SubjPA and SubjSB. | Non effective | Low |
| Seah, 2020, Singapore | Mobile app MapMyFitness (MMF) (developed by Under Armour) | 15, High (girls) | 36 tot (13M, 23F) | 4 weeks | 2-arm | School | PA | Self-Determination Theory | Mobile app (MapMyFitness - MMF) | Not used | Mobile phone | IG MMF app;CG normal lifestyle on weekends PA reported with the pedometer app | Pedometer mobile app Health/Samsung, Health/Pacer Step Counter/Pedometer and Weight Loss Coach. MMF app. | Steps (MMF app). Steps in the weekends (pedometer mobile app Health/Samsung, Health/Pacer Step Counter/Pedometer and Weight Loss Coach). | MVPA, VPA (three-day physical activity recall (3DPAR)) | None | None | Effective on ObjSteps and SubjMVPA/VPA. | Effective | Some concern |
| Smith, 2014, Australia | ATLAS (Active Teen Leaders Avoiding Screen-time) obesity prevention program | 12-14, Middle (at risk of obesity, low income) | 361 tot only male: IG 181; CG 180 | 20 weeks | 2-arm | School | Obesity | Self-Determination Theory, Social Cognitive Theory | Web-based smartphone app + wearable (pedometer for self-monitoring) | teacher professional learning + provision of fitness equipment + researcher-led seminars for students + face-toface PA sessions + lunch-time physical activity leadership sessions run by students + parental strategies for reducing recreational screen-time | Smartphone, wearable (pedometer) | IG ATLAS; CG usual practice (ie, regularly scheduled school sports and physical education lessons). | Accelerometer (Actigraph) | MVPA, CPM (Actigraph GT3X accelerometer) | SB - screen time (modified form of the Adolescent Sedentary Activity Questionnaire - ASAQ) | Fitness - upper body maximal strength (Hand grip dynamom- etry), muscular endurance (90-degree angle push-up test), resistance training skill competency (Resistance Training Skills Battery) | BMI, WC, body fat, Sugar-sweetened beverage consumption, Motivation for school sport | Non effective on ObjMVPA/CPM and SubjSB. | Non effective | Low |
| Stabelini Neto, 2016, Brasil | NA | 8-10, Elementary (obese) | 19 tot (12M, 7F): IG 10, CG 9 | 12 months | 2-arm | School | PA | NA | Wearable (Yamax sw 700 pedometer) | Not used | Wearable (pedometer) | IG pedometer group; CG no intervention | Pedometer (Yamax sw 700) | Steps (Yamax SW 700 pedometer) | None | None | BMI, WC, metabolic parameters | Non effective on Objsteps | Non effective | Some concern |
| Staiano, 2017, USA | NA | 14-18, High (girls, overweight and obese) | 37 tot only females | 12 weeks | 2-arm | Community (dance studio) | PA, screen time, self-efficacy | Social Cognitive Theory | Gamification (Kinect for Xbox 360 gaming console, a television, and the following dancing exergames: Just Dance - Just Dance 3, Just Dance 4, Just Dance 2014, and Just Dance Greatest Hits) and Dance Central (Dance Central 2 and Dance Central 3) | Not used | Console | IG exergaming; CG no exergaming | Accelerometer (Actigraph). Pedometer (Omron GoSmart). | Steps (Omron GoSmart pedometer). VPA, MPA, LPA, Sed (ActiGraph GT3X+ accelerometer). | Screen time, leisure PA (Godin-Shephard Leisure-Time Physical Activity Questionnaire). Exergaming questions (16 multiple choice questionnaire). | NA | BMI, PA self-efficacy, motivation | Non effective on ObjSteps, ObjVPA/MPA/LPA, ObjSB, SubjPA and SubjSB. | Non effective | Some concern |
| Staiano, 2018, USA | GameSquad intervention: Kinect®and Xbox 360®gaming console + a 24-week XboxLive subscription and four exergames (Your Shape:Fitness Evolved 2012,Just Dance 3,DisneylandAdventuresandKinect Sports Season 2) + fitness coach over video chat via theexergame consol | 10-12, Elementary + Middle (overweigh and obese) | 46 tot: IG 23; CG 23 | 24 weeks | 2-arm | Home | Adiposity, cardiometabolic health | Social Cognitive Theory | Gamification (exergame) + telehealth coaching | Booklet with instructions, and to compile for calculate compliance and adherence. | Console | IG GameSquad intervention; CG were asked to maintain their normal level of PA. | Accelerometer (Actigraph; Fitbit Zip) | MVPA, MPA, LPA, Sed (ActiGraph GT3X+ accelerometer). Steps (Fitbit Zip). | PA (7 d between the screening visit and the baseline clinic visit and for 7 d prior to the end-of-study clinic visit with an ActiGraph  GT3X+ accelerometer); | None | Height, weight, resting electrocardiogram, fat mass, resting blood pressure, dietary intake, psychosocial measures | Effective on ObjSteps, ObjMVPA/MPALPA and ObjSB. | Effective | Some concern |
| Thompson, 2016, USA | NA | 14-17, High | 160 tot: IG1 40 (19M, 20F); IG2 40 (15M, 25F); IG3 40 (23M, 17F); CG 40 (20M, 20F) | 12 weeks | 4-arm | Home | PA | Self Determination Theory | Text messaging, wearables (pedometer) | Not used | Smartphone, wearable (pedometer) | IG1 pedometer; IG2 pedometer + goal prompt; IG3 pedometer + goal prompt + theory-informed texts; CG no-treatment. | Accelerometer (Actigraph) | MVPA, steps (Actigraph GT3X+ accelerometer) | None | None | Psychosocial variables, program satisfaction (selfreport questionnaires) | Effective on ObjSteps and ObjMVPA. | Effective | Some concern |
| Trost, 2014, Australia | JOIN for ME program | 8-12, Elementary + Middle | 75 tot (34M, 41F) | 16 weeks | 2-arm | School | PA, Weight | NA | Gamification (exergames: active sports game - Kinect Adventures!; KinectSports;Rare,Mi crosoft Game Studios) | Pediatric weight management program (sessions on foods and drinks, reduction of screen time, goal setting, PA increase. | Console | IG program and active gaming; CG only program | Accelerometer (Actigraph) | MVPA, VPA (ActiGraph GT3X or GT3X+ accelerometer) | PA (daily time spent in MVPA and VPA) | None | BMI | Effective on ObjMVPA/VPA. | Effective | High |
| Tugault-Lafleur,  2023, Canada | Aim2Be app intervention | 10-17 (overweight and obese) | 214 parent-child dyads | 6 months | 2-arm | Home | Health nutrition, PA, screen time) | Social cognitive theory, the player experience and need satisfaction theory, the agency, challenge, uncertainty, discovery, and outcomes framework | Aim2Be app and live coach | Incentive | Mobile phone | IG Aim2Be app+live coach; CG waitlist Aim2Be with no live coach | Accelerometer (Fitbit Flex) | MVPA, steps (Fitbit Flex 2 activity monitor) | PA (PAQ (FOC), PAQ (SF),) SB (SBQ), screen time, fruit and vegetable intake, sugary beverage intake | None | BMI, diet, Screen time | Non effective on ObjMVPA and Objsteps; non effective on Subj PA and SB | Non effective | Low |
| Van Woudenberg, 2018, Netherlands | MyMovez project | 11-14, Middle | 190 tot (49,3%M, 50.7%F): IG1 19 (8M,11F); IG2 74 (32M, 42F); CG 96 (48M, 49F) | NA | 3-arm | School | PA | Theory of Planned Behavior, Self-Determination Theory, Self-Persuasion Theory | Smartphone app + wearable (Fitbit Flex accelerometer, wrist-worn) [=MyMovez Wearable Lab] | Not used | Smartphone | IG smartphone app + wearable; CG no intervention | Accelerometer (Fitbit Flex) | Steps (Fitbit Flex activity monitor) | PA ( number of steps per day with accelerometer Fitbit Flex) | None | None | Non effective on ObjSteps. | Non effective | Some concern |
| van Woudenberg, 2020, Netherlands | MyMovez project | 9-16, Elementary + Middle + High | 446 tot (47%M, 53%F): IG1 131; IG2 123; CG 192 | 5 months | 3-arm | School | PA | Theory of Planned Behavior, Self-Determination Theory, Self-Persuasion Theory | Social network + smartphone app + wearable (Fitbit Flex accelerometer, wrist-worn), or mass media Smartphone app (=MyMovez Wearable Lab) | Not used | Smartphone | IG1 social network intervention; IG2 Mass media intervention; CG no intervention. (All 3 groups used smartphone app and wearable. | Accelerometer (Fitbit Flex) | Steps (Fitbit Flex activity monitor) | PA ( number of steps per day with accelerometer Fitbit Flex) | None | None | Non effective on ObjSteps. | Non effective | Some concern |
| Verswijveren, 2022, Australia | RAW-PA (Raising Awareness of Physical Activity) intervention | 13-18, High (inactive, low income) | 159 tot: IG 75; CG 84 | 12 weeks | 2-arm | School | PA, SB | Social Cognitive Theory, Behavioral Choice Theory | Wearable (Fitbit Flex activity tracker) + social media (FB) + mobile app | Not used | Mobile phone | IG WAT+FB+App; CG waitlist | Accelerometer (ActiGraph GT3X+) | MVPA, LPA, Sed (= time spent in ≤25 counts per epoch) (ActiGraph GT3X+ accelerometer, hip-worn) | None | None | Self-efficacy, peer support, family support, teacher support, self-regulation strategies, perceived barriers to PA, PA enjoyment | Non effective on ObjMVPA, ObjLPA, ObjSed | Non effective | Low |
| Wunsch, 2024, Germany | SMARTFAMILY | Mean 13.3, SD 2.7 | 74 tot (38M, 36F): IG 42, CG 32 | 3 weeks | 2-arm | Home | PA, diet | Self-Determination Theory | Smartphone app | 10 BCTs: behavioral goal setting, prompt review of behavioral goals, prompt self-monitoring of behavior, providing feedback on performance, planning social support or social change, prompt identification as a role model or position advocate, setting graded tasks, shaping, prompt rewards contingent on effort or progress toward behavior, and providing rewards contingent on successful behavior. | Smartphone | IG smartphone app (SMARTFAMILY (SF) app); CG received no material and was not contacted during the intervention | Accelerometer (Movisens Move 3 and Move 4) | MVPA, steps (Movisens Move 3 and Move 4 accelerometer, right side hip-worn) | MVPA (60-Minute Screening Measure); PA (PA diary) | None | BMI, diet (F&V consuption), perceived general health, intrinsic motivation for PA and eating behavior, joint PA and Meals Within the Family | Non effective on ObjMVPA, ObjSteps, SubjPA | Non effective | Low |
