# Supplementary material for: Digital Tools’ Effectiveness on Physical Activity Outcomes in Children and Adolescents: Umbrella Review
Source: JMIR Public Health Surveill. 2026 Mar 24;12:e75769. doi: 10.2196/75769 (PMC13013097; doi:10.2196/75769)
Supplement: Multimedia Appendix 6 — Risk of bias of the selected randomized controlled trials. [file publichealth-v12-e75769-s006.pdf]

First author, publication year	Randomisation process	Deviations from the intended interventions	Missing outcome data	Measurement of the outcome	Selection of the reported result	Overall
Allafi, 2020	!	+	+	+	!	+
Babic, 2016	!	-	!	!	!	!
Baldursdóttir, 2017	+	!	+	!	+	+
Baranowski, 2011	!	!	+	+	+	+
Baranowski, 2012	!	!	-	!	!	!
Baranowski, 2019	!	!	+	+	+	!
Brannon, 2017	!	-	+	!	!	!
Caillaud, 2022	!	-	!	!	!	!
Carlin, 2021	+	!	!	+	+	+
Chen, 2011	!	!	+	!	+	!
Comeras-Chueca, 2022	!	!	+	+	!	!
Dewar, 2013	!	!	-	+	+	!
Dewar, 2014	!	-	+	+	+	+
Direito, 2015	+	+	+	+	+	+
Duncan, 2010	!	-	+	!	-	-
Duncan, 2024	+	!	+	+	!	+
Errickson, 2012	!	!	-	+	-	-
Ezendam, 2012	!	!	+	!	!	!
Garde, 2015	+	+	+	+	!	!
Garde, 2016	+	!	+	+	+	+
Garde, 2018	+	!	+	+	+	+
Graves, 2010	!	!	-	!	-	-
Guagliano, 2020	+	!	+	+	+	+
Guthrie, 2015	!	!	+	+	!	!
Honas, 2024	+	!	+	+	!	+
Jake-Schoffman, 2018	+	!	+	+	!	+
Jauho, 2015	+	!	-	+	!	!
Lau, 2016	!	-	-	+	!	-
Layne, 2022	+	!	+	+	+	+
Lee, 2012	+	-	!	+	+	+
Leinonen, 2017	+	+	!	+	!	+
Lubans, 2011	!	-	+	!	!	!
Lubans, 2012	!	-	+	!	!	!
Lubans, 2016	+	-	+	!	!	!
Maddison, 2011	+	+	-	+	!	+
Maloney, 2012	+	-	-	!	!	-
Manley, 2014	!	!	+	!	!	!
Morgan, 2014	+	-	+	+	!	+
Morgan, 2019	+	-	+	+	!	!
Morgan, 2022	+	-	+	+	!	!
Morris, 2019	+	!	-	+	+	!
Ngo, 2014	!	-	+	+	+	!
Petrušič, 2022	+	!	+	+	!	+
Pfeiffer, 2019	!	+	+	+	!	+
Pope, 2018	!	-	+	!	+	!
Rhodes, 2017	!	!	+	!	!	!
Ridgers, 2021	+	!	+	+	!	+
Robbins, 2019	!	!	+	!	!	!
Robertson, 2018	+	-	-	+	!	-
Ruotsaleinen, 2015	+	+	+	!	!	+
Seah, 2021	!	-	+	!	!	!
Smith, 2014	+	+	+	+	!	+
Stabelini Neto, 2016	+	!	-	!	!	!
Staiano, 2017	!	-	+	+	!	!
Staiano, 2018	!	!	+	!	!	!
Thompson, 2016	+	!	+	+	-	!
Trost, 2014	!	-	-	+	-	-
Tugault-Lafleur, 2023	+	!	+	+	!	+
Van Woudenberg, 2018	!	+	+	+	!	!
van Woudenberg, 2020	!	+	+	+	!	!
Verswijveren, 2022	+	-	+	+	+	+
Wunsch, 2024	+	-	+	+	+	+
